# Discovery of a Low Thermal Conductivity Oxide Guided by Probe Structure Prediction and Machine Learning

**DOI:** 10.1002/anie.202102073

**Published:** 2021-06-17

**Authors:** Christopher M. Collins, Luke M. Daniels, Quinn Gibson, Michael W. Gaultois, Michael Moran, Richard Feetham, Michael J. Pitcher, Matthew S. Dyer, Charlene Delacotte, Marco Zanella, Claire A. Murray, Gyorgyi Glodan, Olivier Pérez, Denis Pelloquin, Troy D. Manning, Jonathan Alaria, George R. Darling, John B. Claridge, Matthew J. Rosseinsky

**Affiliations:** ^1^ Department of Chemistry University of Liverpool Crown Street Liverpool L69 7ZD UK; ^2^ Leverhulme Research Centre for Functional Materials Design The Materials Innovation Factory University of Liverpool 51 Oxford Street Liverpool L7 3NY UK; ^3^ Diamond Light Source Harwell Science and Innovation Campus Oxfordshire OX11 0DE UK; ^4^ University of Manchester Dalton Cumbrian Facility Westlakes Science Park Moor Row CA24 3HA UK; ^5^ Laboratoire CRISMAT ENSICAEN 6 boulevard du Maréchal Juin 14050 Caen Cedex 4 France; ^6^ Department of Physics University of Liverpool Oxford Street Liverpool L69 7ZE UK

**Keywords:** aperiodic structure, machine learning, metastable compounds, thermal conductivity, titanium oxides

## Abstract

We report the aperiodic titanate Ba_10_Y_6_Ti_4_O_27_ with a room‐temperature thermal conductivity that equals the lowest reported for an oxide. The structure is characterised by discontinuous occupancy modulation of each of the sites and can be considered as a quasicrystal. The resulting localisation of lattice vibrations suppresses phonon transport of heat. This new lead material for low‐thermal‐conductivity oxides is metastable and located within a quaternary phase field that has been previously explored. Its isolation thus requires a precisely defined synthetic protocol. The necessary narrowing of the search space for experimental investigation was achieved by evaluation of titanate crystal chemistry, prediction of unexplored structural motifs that would favour synthetically accessible new compositions, and assessment of their properties with machine‐learning models.

## Graphical abstract

Ba_10_Y_6_Ti_4_O_27_ has a complex aperiodic structure with the lowest thermal conductivity of any first‐transition‐series oxide. It is metastable, which makes it challenging to identify. Experimental exploration was focused sufficiently by structure prediction and property‐targeted machine learning to enable the isolation of this material (see picture), whose structural motifs provide new insight into the control of heat transport.

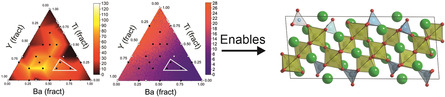

## Introduction

New directions in science and technology are opened by the discovery of materials with previously unknown structures that confer leading properties.^[1]^ The initial identification of such lead materials is challenging because both the properties and the stability of a new material are determined by its structure and its composition in concert, neither of which can be known at the outset.^[2]^ The vast chemical space impedes selection of composition,^[3]^ while the absence of bounds on unit cell metrics and dimensionality^[4]^ obstructs identification of structure. Here we tackle this challenge by fusing the prediction of unexplored structural motifs that will provide experimentally accessible new compositions with assessment of their properties by machine learning. This enables the isolation of a metastable aperiodic oxide that produces the lowest reported thermal conductivity for a first transition series oxide. The outperformance of this new lead material is conferred by the unique structure, which adds distinctive property‐controlling motifs. This strategy for exploration beyond our knowledge frontier of stable compositions, guided by evaluation of the properties within it, narrows the space of chemistry to those regions where outperforming functional materials are located.

Although there are large numbers of stable and metastable materials (over 200 000 crystalline inorganic materials are reported in the Inorganic Crystal Structure Database^[5]^), their occurrence relative to the size of the relevant composition space^[3]^ is infrequent. There is no lower bound to the discretization needed to cover the space fully as elemental ratios are not formally limited. Chemical space is larger even than composition space as it includes structure: one composition can have multiple polymorphs, there is no upper bound to the unit cell metrics that define periodic structures, and the number of spatial dimensions needed to describe such structures can extend beyond three. Further, the determination of synthetic protocols to isolate materials with new chemistry, their processing to the form required for property measurement and the understanding of their structures over the relevant length scales are all nontrivial. Together, these considerations demonstrate the need for efficient selection of the most promising regions of composition space to investigate for new leads, where property performance near or beyond that of the best‐in‐class materials arises from previously unknown structures. CsBi_4_Te_6_, which opened a new class of low thermal conductivity materials for applications as cryogenic thermoelectric materials,^[6]^ is an example lead material.

In this work, we locate leads that are missing within previously explored chemical space. Thermal conductivity, *κ*, is of interest both for application (e.g., low *κ* in thermal barrier coatings^[7]^ and thermoelectric materials^[8]^) and as a fundamental property of a material that is directly connected to its structure and bonding via phonon mobility.^[9]^ As the ternary titanium oxide SrTiO_3_ is the parent of leading n‐type oxide thermoelectric materials for high temperature waste heat harvesting,^[10]^ the enhanced structural diversity of the quaternary titanate spaces accessed by adding the smaller and more highly charged Y^3+^ to Sr^2+^ and the yet larger Ba^2+^ offer a route to low *κ* oxides. Extensive, well‐executed studies of this chemistry have led to the construction of thermodynamic models^[11]^ that are consistent with the existence of the single reported quaternary Ba_6_Y_2_Ti_4_O_17_.^[12]^ We tested this understanding by prioritising the experimental exploration of new compositions predicted to offer stable unexplored structures with machine learning of their properties.

## Results and Discussion

**Figure 1:**
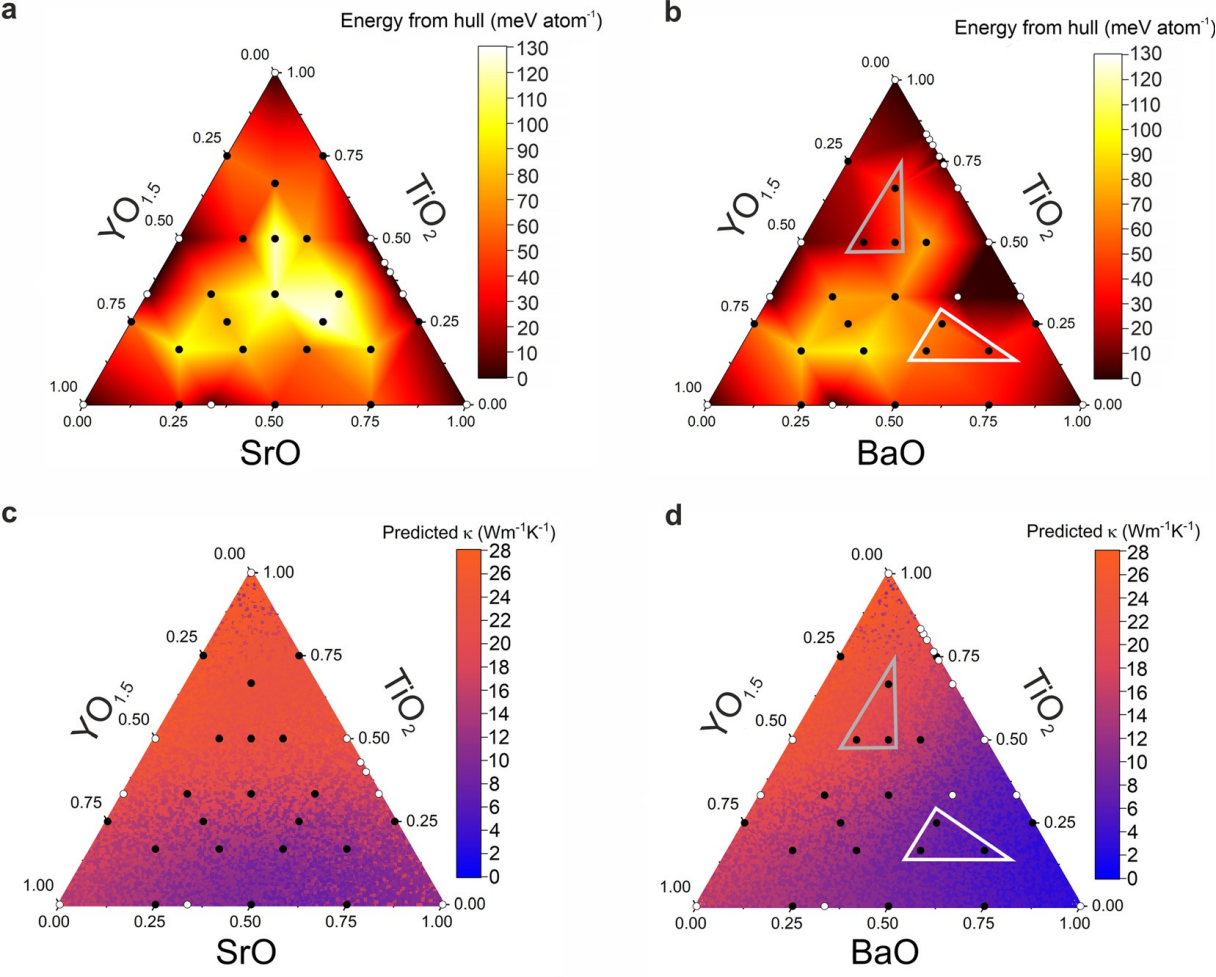
Probe structure stability and machine learning property prediction for the two phase fields. a,b) Convex hulls computed with DFT using probe structures for Y^3+^–Sr^2+^–Ti^4+^–O^2−^ (a) and Y^3+^–Ba^2+^–Ti^4+^–O^2−^ phase fields (b). c,d) Predicted thermal conductivity, *κ*, from the 64×2 neural network machine learning model for Y^3+^–Sr^2+^–Ti^4+^–O^2−^ (c) and Y^3+^–Ba^2+^—Ti^4+^‐O^2−^ (d). Black points indicate compositions at which probe structures were generated, white points indicate known phases. The two outlined triangles in (b) and (d) indicate the low‐energy regions in the phase field likely to contain new synthetically accessible phases, and the white triangle in (b) and (d) indicates the compositional target region identified as most likely to contain new phases with low thermal conductivity. The average predicted thermal conductivity of the white triangular region (7±2 Wm^−1^ K^−1^) is lower than that of the grey (16±3 Wm^−1^ K^−1^).

Calculation of the energies of probe structures^[13]^ (hypothetical structures with unit cells large enough to sample the chemical bonding and energies accessible at a given composition in order to target synthetic effort, generated here with FUSE^[13]^ and MC‐EMMA^[14]^), allows the identification of low‐energy regions of composition space that yield experimentally isolable new phases with previously unknown structures.[^13^, ^14^] A probe structure investigation of the Y^3+^–Sr^2+^–Ti^4+^–O^2−^ phase field computed with density functional theory (DFT), previously evaluated only with force fields,^[13]^ indicates that no low‐energy regions corresponding to such candidate phases exist (Figure 1 a). We constructed a similarly detailed DFT map of the probe structure energies for quaternaries in the Y^3+^–Ba^2+^–Ti^4+^–O^2−^ field, using the MC‐EMMA method to expand the compositions previously studied.^[14]^ This, in contrast to the Sr case, identifies two low‐energy regions (shown as bold outlined grey and white triangles in Figure 1 b) where a set of new compositions are sufficiently close to the convex hull (the energy surface defined by stable compounds:^[15]^ stable defines phases that are thermodynamically stable relative to each of the other phases in the field from zero Kelvin DFT calculations) to motivate synthesis; the five lowest energy compositions (not including the known phase Ba_0.5_Y_0.167_Ti_0.333_O_1.417_, which is on the convex hull, Figure 1 b) being Ba_0.167_Y_0.333_Ti_0.5_O_1.667_ (+19 meV atom^−1^ from the convex hull), Ba_0.667_Y_0.167_Ti_0.167_O_1.25_ (+42 meV atom^−1^), Ba_0.167_Y_0.167_Ti_0.667_O_1.75_ (+50 meV atom^−1^), Ba_0.5_Y_0.333_Ti_0.167_O_1.333_ (+61 meV atom^−1^) and Ba_0.5_Y_0.25_Ti_0.25_O_1.375_ (+62 meV atom^−1^; Table S1).

Machine learning models were used to determine the priority for experimental exploration by comparing the likely thermal conductivity of compositions in both phase fields in the context of other oxides. While probe structures allow evaluation of candidate materials in unknown composition space, machine learning models are generated by interpolation of existing knowledge in the training data. Machine learning models for thermal conductivity were constructed using experimental and computational data from Gaultois et al.^[16]^ and TEDesignLab^[17]^ to predict *κ* solely from the composition. This differs from previous models in the literature which incorporate features from the crystal structure to predict lattice thermal conductivity.[^18^, ^19^] The role of the models here is in relative ranking of the candidate compositional regions emerging from the probe structure calculations, rather than precise prediction of the thermal conductivities of individual compositions. As the structure of the target compositions is not known, only models based upon composition were considered, which, while less accurate than those also using structure, match the nature of the discovery task. These models are similar to composition‐only models based on the binary classification of whether a material potentially has thermal conductivity below a given threshold (i.e., <10 Wm^−1^ K^−1^)[^20^, ^21^] but are built here as regression models to identify continuous trends in thermal conductivity with composition and thus locate composition regions where the lowest values may be experimentally obtained.

Initially nine models were trained using various algorithms of differing complexity (Table S2). We used the R^2^ and mean squared error (MSE) to assess performance and thus concluded that neural network and random forest algorithms were most suited to this work (R^2^>0.65, MSE<19), as they clearly outperformed simpler algorithms (R^2^<0.55, MSE>24). Models trained using random forests and neural network algorithms provide consistent results with no significant differences in their performance (Table S3, Figure S1) and were used to predict the thermal conductivities within the chosen phase fields (Figure 1 c,d); final models used the featurizer from Matminer (accessed August 2019), and the final feature vectors contained 121 features. These models indicate that only the Y^3+^–Ba^2+^–Ti^4+^–O^2−^ phase field is a likely source of low thermal conductivity oxides (based on the probe structure calculations in Figure 1 b and the thermal conductivities in Figure 1 d), and prioritizes one of the two low‐energy regions in this field as more likely to afford a low thermal conductivity (these regions are shown in Figure 1 d with the same triangle representation used in the energy calculations in Figure 1 b). Specifically, the unexplored region of composition space defined by the white triangle Ba_0.667_Y_0.167_Ti_0.167_O_1.25_–Ba_0.5_Y_0.333_Ti_0.167_O_1.333_–Ba_0.5_Y_0.25_Ti_0.25_O_1.375_ (Figure 1 b,d) has a much lower average predicted thermal conductivity, and emerges as a suitable candidate in which to isolate new lead compounds with low thermal conductivities, despite lying within a previously studied phase field.

**Figure 2:**
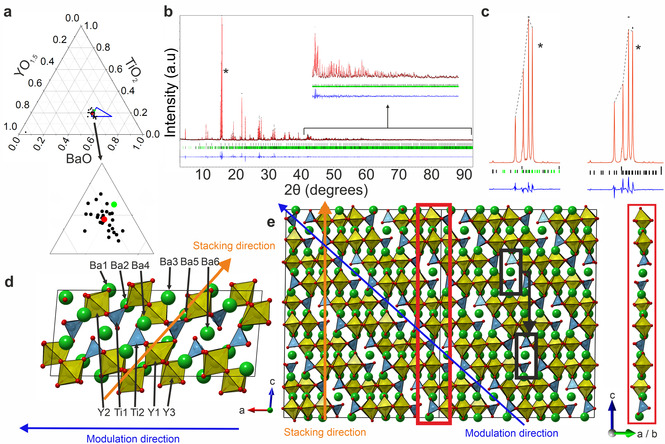
Isolation and structure solution of Ba_10_Y_6_Ti_4_O_27_. a) TEM–EDX measurement of 29 particles from a sample at the phase pure nominal composition Ba_10_Y_6_Ti_4_O_27_, identified by synthesis at the compositions shown in Figure S2, yielded the mean ratio Ba_0.50(3)_Y_0.31(2)_Ti_0.19(2)_O_1.35(10)_ (Ba_10.0(6)_Y_6.2(1)_Ti_3.8(1)_O_27(2)_), marked in red; O content based on Ba^2+^, Y^3+^, Ti^4+^ and O^2−^, the compositional target region from Figure 1 b,d is shown in blue. b) Rietveld refinement yields the composition Ba_0.5_Y_0.277(8)_Ti_0.223(8)_O_1.35_ (Ba_10_Y_5.54(16)_Ti_4.46(16)_O_27_, marked in green in (a)). Upper tick marks indicate reflections from Y_2_O_3_ (1.68(4) mol %), lower tick marks Ba_10_Y_6_Ti_4_O_27_ (black: fundamental subcell reflections; green: satellites from structural modulation; blue: difference between calculated and observed). c) The most intense reflections from Ba_10_Y_6_Ti_4_O_27_, marked with “*” in (b), refined in aperiodic (left) and commensurate (right) models (full commensurate fit shown in Figure S4). On the left, the indices for the major component of each reflection are (the indices have four components: *h*, *k*, *l* as normal and *m* to indicate the satellite order, where 0 indicates a reflection from the subcell only), from left to right, [1022] +[202−2], [0200], [013−1] (the most intense reflection in the diffraction pattern) and [−2111]. Dashed lines guide the eye between the calculated intensities. d) 4×1×1 supercell of the aperiodic model at *t*=0, with the perovskite stacking direction in orange and the modulation direction in blue, viewed perpendicular to *a*. e) View along [110] of the pseudo‐cubic representation of the structure (see the Supporting Information), showing the 10 polyhedron motif (red box), which shifts between stacks as shown by the pair of tetrahedra within the black box. Ba green, Y yellow, Ti cyan, O red.

Initial synthesis focused on this target region at the two compositions Ba_0.667_Y_0.167_Ti_0.167_O_1.25_ and Ba_0.5_Y_0.333_Ti_0.167_O_1.33_ (which are shown within the white triangle in Figure 1 b,d) over a range of synthesis conditions (described in the Supporting information, Figure S2). Powder X‐ray diffraction (PXRD) analysis then revealed Bragg reflections beyond those expected from database and literature reports on the Y^3+^–Ba^2+^–Ti^4+^–O^2−^ phase field (Figure S2). TEM–EDX analysis of these two multiphasic samples indicated a phase with the previously unreported composition Ba_0.50(4)_Y_0.30(3)_Ti_0.20(2)_O_1.35(13)_, within the target region (Figure S2a). An additional 30 compositions were then sampled to isolate this new phase (Figure S2), which is metastable (Figure S3) and therefore simultaneously required detailed optimization of synthetic conditions (reaction time, temperature and cooling rate; see the Supporting information) to isolate a high purity white sample of nominal composition Ba_0.53_Y_0.29_Ti_0.18_O_1.32_. The measured TEM–EDX composition (Figure 2 a) of Ba_0.50(3)_Y_0.31(2)_Ti_0.19(2)_O_1.35(10)_ (normalised to Ba_10.0(6)_Y_6.2(4)_Ti_3.8(4)_O_27(2)_, hereafter referred to as Ba_10_Y_6_Ti_4_O_27_) is within 1 e.s.d. of nominal.

**Figure 3:**
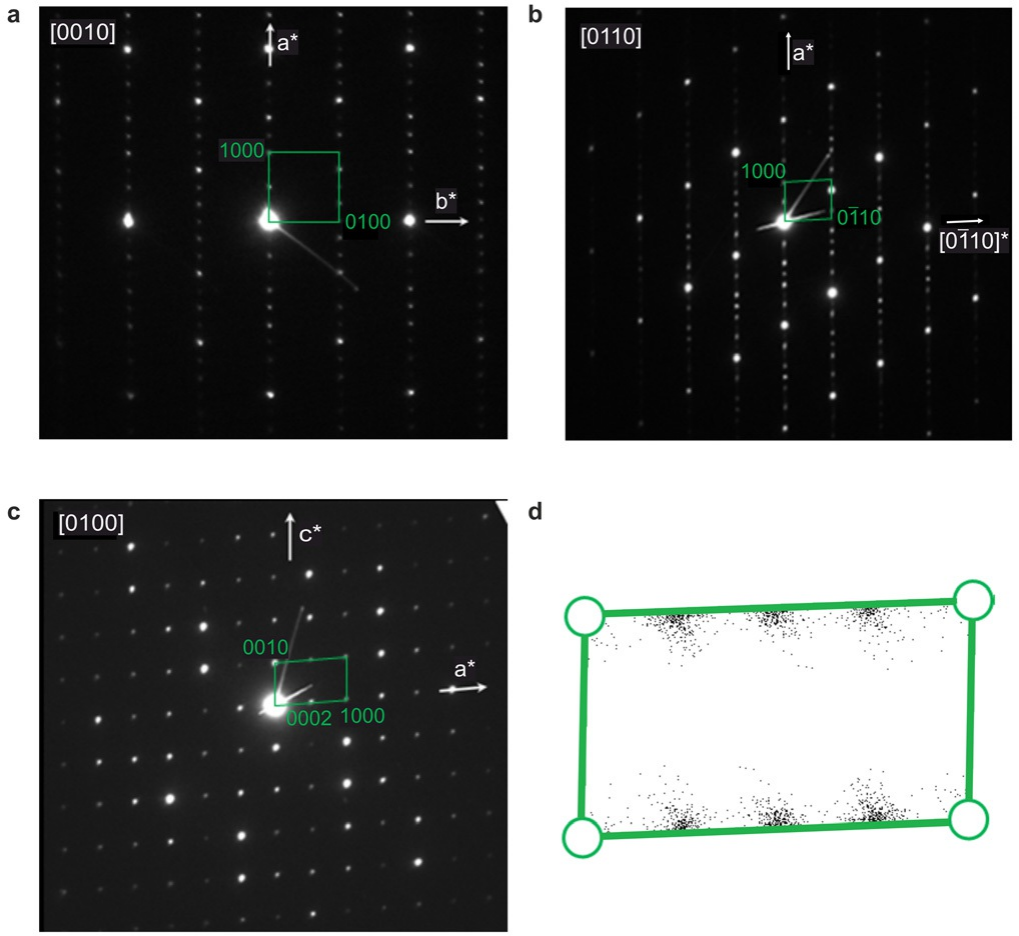
Electron diffraction of Ba_10_Y_6_Ti_4_O_27_. a–c) Experimental diffraction (PEDT) patterns of the [0010] (a), [0110] (b) and [0100] zone axes (c); note the *m*=2 *n* reflection condition means only even satellites are observed. d) Precession electron diffraction projected onto the a* c* plane; the dispersed groups of supercell reflections result in the diffraction pattern only being adequately indexed with a (3+1)D cell.

The unit cell and space group of the new quaternary Ba_10_Y_6_Ti_4_O_27_ were determined by precession electron diffraction (Figure 3) after attempts from PXRD were unsuccessful, then refined (Figure 2 b) against high Q resolution synchrotron PXRD data. Ba_10_Y_6_Ti_4_O_27_ is aperiodic with the unit cell: *a*=*x*_1_=6.20543(3) Å, *b*=*x*_2_=6.10136(3) Å, *c*=*x*_3_=10.42917(5) Å, *β*=94.8316(3)° and **q**=*x*_4_*=0.24964(2) a*−0.00021(4) c* in the (3+1)D superspace group *P*2/*m*(a0g)0s (where *x_n_* are the unit cell vectors in higher dimensional space and *x*, *y*, *z* and *t* are their internal coordinates). The strong intensity of the satellite reflections (Figure 2 b,c) signals the discontinuous crenel‐type variation of the site occupancies^[22]^—the most intense reflection is in fact a satellite rather than from the subcell. Given the difficulty in solving (3+1)D structures with very strong satellite peaks we first attempted a solution to the commensurate approximant.^[23a]^

**Figure 4:**
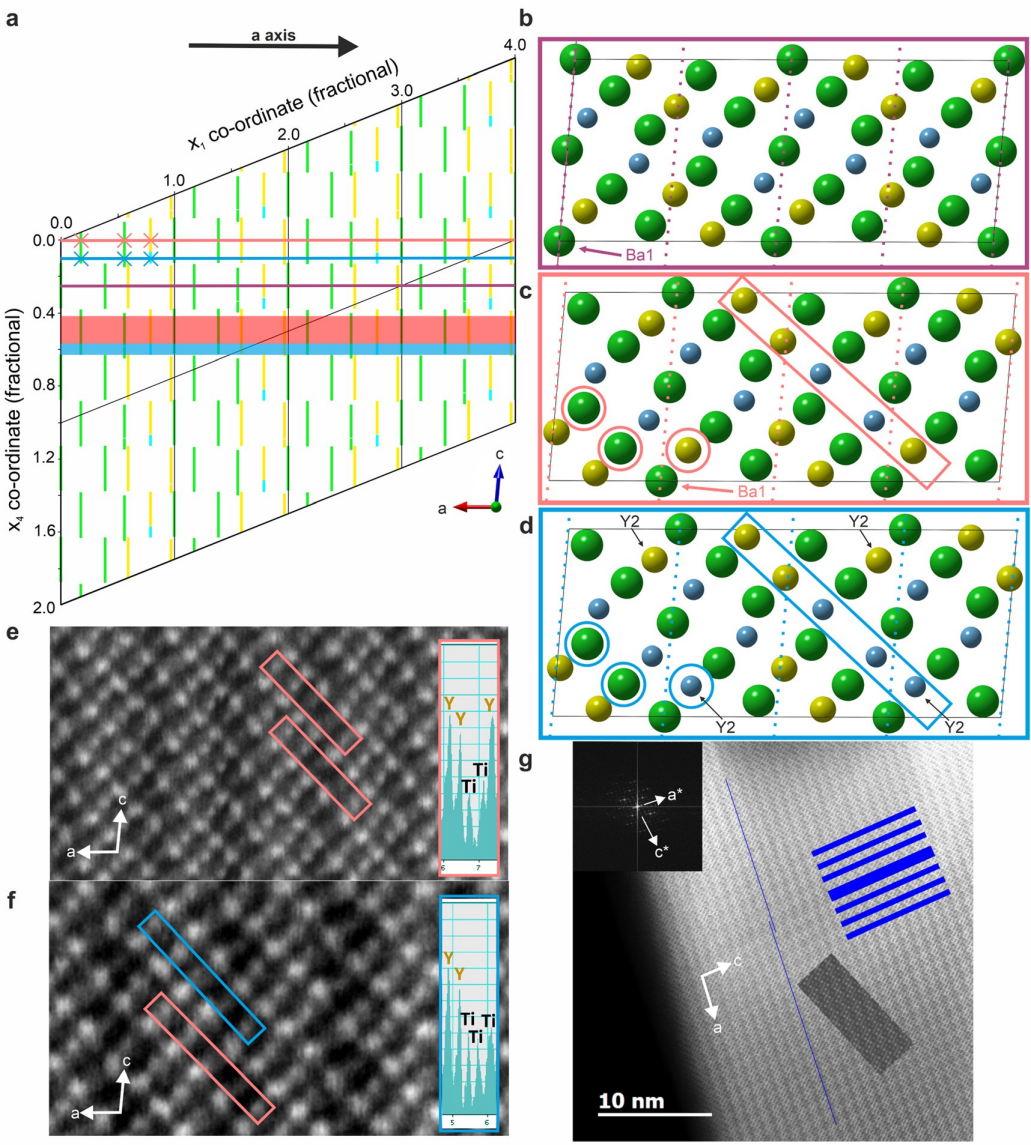
Aperiodic structure of Ba_10_Y_6_Ti_4_O_27_. a) Simplified x_1_ versus x_4_ projection of Ba_10_Y_6_Ti_4_O_27_ (*z*=0−1/3, *y*=0−1; full version in the Supporting Information), showing crenel functions describing the occupancies of the Y and Ba sites only; barium crenels are green, yttrium are yellow and regions of titanium for yttrium substitution cyan. External space is represented by sections perpendicular to x_4_ and atoms are observed when these intersect crenels—the real space direction shown here is *a*. Three real space sections with origins *t*=1/4 (purple), *t*=0 (pink) and *t*=0.1 (blue) are marked. The corresponding approximants (b–d) are shown projected onto the *ac* plane, anions are omitted for clarity (Ba green, Y yellow, Ti cyan). Intersections on the *a* direction are marked with crosses in (a) and the corresponding atoms circled in (c) and (d). The structure does not vary significantly within the single‐atom occupancy regions of the crenel, as there are few positional modulations—the pink and cyan shading in (a) marks regions of *t* that produce structures very similar to those at *t*=0 (ca. 60 % in (c)) and *t*=0.1 (ca. 20 % in (d)), where half the Y2 sites are substituted by Ti (all four Y2 sites are labelled in (d)). At *t*=−0.1 (ca. 20 %; see the Supporting information), the remaining Y2 sites are substituted. Dotted lines in (b–d) indicate the subcell of the modulated structural model. e) HAADF micrograph of a region typical of *t*=0 with a representative line scan showing the Ti–Y–Y–Y–Ti repeat (pink boxes), matching the model in (c), where a pink box highlights the five B sites. f) HAADF micrograph of a region typical of *t*=0.1 with a representative line scan showing the Ti–Y–Y–Y–Ti repeat from the area shown in the pink box (as in (e)) and the Ti–Ti–Y–Y–Ti repeat from the area in the blue box, which matches the model in (d), where a blue box highlights the five B sites. g) Micrograph of the region associated with the interface between the shifted versions of the structure at *t=*0 and *t*=1/4 shown in (c) and (b), illustrated by the label for the Ba1 site. Note the thicker region of dark contrast (highlighted with blue boxes) and kink in the perpendicular dark stripes highlighted by the thin blue lines, both arising from the introduction of extra atomic layers associated with the aperiodicity and origin shifts shown in (b) and (c). Insets show the Fourier transform of the whole image, which is consistent with the aperiodic structural model, rather than multiple diffraction patterns that might be expected if there were twinned, shear, defect or intergrowth regions.

The periodic approximation of the structure of Ba_10_Y_6_Ti_4_O_27_ adopts an ABO_3−*x*_ perovskite‐related structure with a new 10a_p_ stacking sequence (a_p_ denotes one perovskite unit, Figure 2 d,e), described in terms of the B‐site polyhedra as O–O–T_t_–T_t_–O–O–T_c_–O–O–T_c′_, where O, T_t_, T_c_ and T_c′_ indicate octahedra, terminal tetrahedra and two orientations of corner‐sharing tetrahedra, respectively. This structural unit combines a known defect perovskite sequence with a brownmillerite‐related stacking that features an unusual relationship between the two tetrahedral orientations. Although **q** is very close to the commensurate **q_comm_** =1/4 a*−0 c*, the aperiodic model affords a superior refinement (Figure 2 c), where the structure is periodic in (3+1)D superspace but aperiodic in the observed 3D projection, and predicts the real space images in Figure 4 e–g.

As all site occupancies are defined by discontinuous functions, the structure can also be described as a quasicrystal, as for the 4D quasicrystal case there is an equivalent (3+1)D description, related by an alternative choice of axes,^[23a]^ that we use here.^[23b]^ Ref. [23a] (pp. 71 and 82) shows that a 4D quasicrystal is equivalent to a (3+1)D structure with discontinuous occupancy domains. This is the case we refine here, hence our proposal of the quasicrystal nature of Ba_10_Y_6_Ti_4_O_27_. This is analogous to the 4D alloy quasicrystals in Ref. [24], which have discontinuous occupancy domains and no non‐crystallographic rotations (no 4D quasicrystal reported currently has non‐crystallographic rotations). Oxide quasicrystals have been observed at interfaces,[^25^, ^26^, ^27^] however, the material presented here is the first that has been proposed as a quasicrystal in the bulk. Given that most attempts to identify quasicrystals have been associated with forbidden symmetries, further examples may emerge from appropriate evaluation of the structures of known compounds against these criteria. Refinement against synchrotron diffraction data shows that periodic substitution at the Y2 site by Ti, and separation of the Ba5 and Y1 sites into two components (the minority sites have positions which modulate about the average position), occurs in 40 % of the material in this coherent fashion (Supporting information).

The crenel functions (Figure S5) at the Y and Ba sites are shown in Figure 4 a in the x_1_ x_4_ plane. Real space is represented by sections perpendicular to x_4_. As x_4_=**q.r**+t (where **r** is a lattice translation of the subcell, defined by x_1_, x_2_ and x_3_), in the commensurate case (**q_comm_**), only four points along t will be visited, (t, t+1/4, t+1/2 and t+3/4), whereas in the actual aperiodic case, all points are visited as **q.r** is irrational. Whilst the structure is no longer periodic in external space, it is periodic in superspace. We can therefore visualize local arrangements of atoms in real space by examining supercells with different origins in t along x_4_. Three representative sections are shown in Figure 4 a. For approximately 60 % t, the real space projection will look like that at *t*=0 (Figures 4 c and 2 d), as **q** is very close to 1/4 a*, and we observe regions closely resembling this approximant by TEM for most of the sample (Figure 4 e). The replacement of part of the Y2 site crenels with titanium creates coherent regions where the Y2 B sites are occupied by Ti, as in the section *t*=0.1 in Figure 4 d and *t*=−0.1 (Figure S6)—charge balancing oxygen atoms are expected to replace the BaO layer associated with the terminal TiO_4_ with a BaO_3_ layer,^[28]^ but, at <1 % occupancy across the whole structure, are not refinable. These regions are also observed in the HAADF images, Figure 4 f, corresponding to approximately 40 % of the structure.

As **q**≈(1/4 −δ) a*, the superspace model predicts that as we continue along external space, we also move slowly along *t* such that the effective origin of the next supercell will not be at *t*, but *t*−4δ. We then expect to see wide bands of the *t*=0 and *t*=±0.1 structures repeating on a micron length scale, as observed in TEM. With *δ*≈0.0002, the origin will shift by 1/4 in *t* after approximately 1250 repeats of the supercell, as in Figure 4 b, where the associated shift in the cation columns from Figure 4 c is apparent. We then expect to generate a boundary between these shifted structures on moving between Y/Ti crenels and Ba ones along t, which is also observed by microscopy (Figures 4 g and S6).

**Figure 5:**
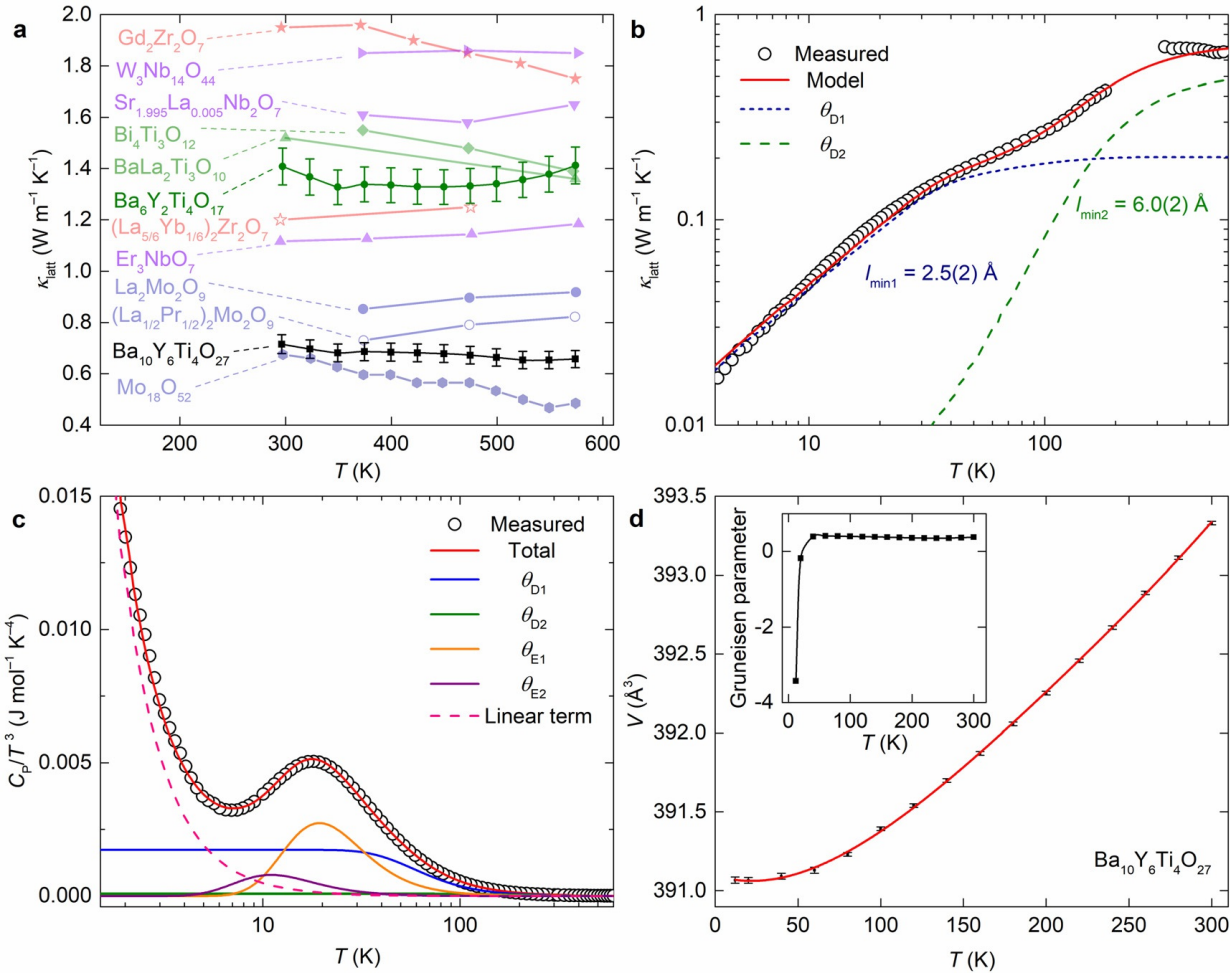
Thermal properties of Ba_10_Y_6_Ti_4_O_27_. a) Comparison of oxide materials with *κ*_latt_ below 2 Wm^−1^ K^−1^ against Ba_10_Y_6_Ti_4_O_27_ (black):[^31^, ^32^, ^33^, ^34^, ^35^, ^36^, ^37^, ^38^, ^39^] niobates (purple), zirconates (red), molybdates (blue), titanates (green), including the previously reported quaternary Ba_6_Y_2_Ti_4_O_17_ measured as part of this study. The outperformance of Ba_10_Y_6_Ti_4_O_27_ is clear through further comparison based on simple structural parameters shown in Figure S9 b) Measured thermal conductivity (*κ*_latt_) of Ba_10_Y_6_Ti_4_O_27_ shown against a modified Debye–Callaway model with contributions from two Debye temperatures (*θ*_D1_ and *θ*_D2_), highlighting the phonon glass behavior. c) Fit to heat capacity (*C*_P_/*T* ^3^ vs. *T*) of Ba_10_Y_6_Ti_4_O_27_, highlighting the modes needed to model the low temperature boson peak, and the linear term (Figure S7). d) Unit cell volume (*V*) of Ba_10_Y_6_Ti_4_O_27_ as a function of temperature with fitted polynomial (*R* ^2^=0.9998, Table S4). The Grüneisen parameter (inset) is extracted from these data.

The choice of phase field was qualitatively validated by measurement of the thermal conductivity of the reported quaternary Ba_6_Y_2_Ti_4_O_17_, which proved to be low (Figure 5 a), supporting the selection of quaternary titanates with Ba instead of Sr. The key distinguishing structural feature of the new quaternary Ba_10_Y_6_Ti_4_O_27_ from both the training set used to build the ML model and the known quaternary is its four‐dimensional structure, aperiodic but long‐range ordered in three dimensions, which in light of the low reported thermal conductivities for intermetallic quasicrystal metals, aperiodic intermetallics and degenerate semiconductors, *κ*_latt_,^[29]^ can be expected to confer low thermal conductivity on the new quaternary. This motivated the processing of the new phase Ba_10_Y_6_Ti_4_O_27_ to the density required for measurement (Methods).

Processing Ba_10_Y_6_Ti_4_O_27_ is challenging owing to its metastability (Figure S3) and the high melting temperatures of titanium oxides. Accordingly, in order to produce specimens suitable for measuring thermal conductivity, samples were densified by spark plasma sintering at 593 K under 800 MPa uniaxial pressure. The lattice thermal conductivity of Ba_10_Y_6_Ti_4_O_27_ increases monotonically with temperature, reaching a value of 0.715(36) W m^−1^ K^−1^ at 298 K (Figure 5 b). This measured thermal conductivity is the lowest reported for any first transition series metal oxide over a wide temperature range comparable with the lowest second transition series oxide Mo_18_O_52_ (Figure 5 a)[^31^, ^32^, ^33^, ^34^, ^35^, ^36^, ^37^, ^38^, ^39^] and the temperature dependence of *κ*_latt_ for Ba_10_Y_6_Ti_4_O_27_ indicates that phonon transport is glasslike, where a solid behaves as a disordered material with respect to phonons.^[40]^ In particular, the peak that is typical of well‐ordered crystalline systems is not observed (Figure 5 b). Similar behaviour is observed in the low temperature variation of *κ*_latt_ in other aperiodic materials[^41^, ^42^, ^43^] and in some quasicrystal materials[^44^, ^45^, ^46^, ^47^] in which the aperiodicity of the lattice increases phonon scattering from extended structural motifs and generates Anderson localization.^[47]^ The phonon‐glass behaviour of Ba_10_Y_6_Ti_4_O_27_ is supported by the observation of excess specific heat at low temperatures (often referred to as a boson peak, Figure 5 c), which arises from highly localized vibrations.[^48^, ^49^] The heat capacity of Ba_10_Y_6_Ti_4_O_27_ is modelled with a linear term, two Einstein temperatures (*θ*_E1_=95.5(5) K, *θ*_E2_=54.3(4) K) and two Debye temperatures (*θ*_D1_=290(2) K, *θ*_D2_=800(10) K; Figure S7), consistent with phonon calculations and Raman spectra (Figure S8a) revealing low‐frequency modes from ionic, high Ba atomic mass, low‐force‐constant Ba−O bonds in *θ*_D1_, and high‐frequency contributions of *θ*_D2_ from more covalent bonding in TiO_x_ polyhedra. The linear contribution to the heat capacity (Figure 5 c), which is unexpected for an insulator (Ba_10_Y_6_Ti_4_O_27_ band gap of 4.179(3) eV, Figure S8b) and would be masked by the electronic contribution in intermetallics,^[50]^ demonstrates the vibrational tunnelling states in Ba_10_Y_6_Ti_4_O_27_.[^50^, ^51^] The presence of the boson peak and linear contribution in the heat capacity are characteristic thermal properties of glasses and have been proposed to be a general feature of aperiodic materials as well.^[52]^ The speed of sound (*v*_s_) for Ba_10_Y_6_Ti_4_O_27_ of 2204(45) m s^−1^, obtained from Grüneisen parameters (Figure 5 d) is, like the thermal conductivity, very low for an oxide, approximately half that of known quaternary Ba_6_Y_2_Ti_4_O_17_ (4443(21) m s^−1^).

The thermal conductivity of Ba_10_Y_6_Ti_4_O_27_ is described well by a modified Debye–Callaway model^[53]^ (Figure 5 b) that includes the experimentally determined *θ*_D1_, *θ*_D2_, *θ*_E1_ and *v*_s_ Table S5). The minimum phonon mean free paths (*l*_min_) of 2.5(2) Å and 6.0(2) Å for *θ*_D1_ and *θ*_D2_ confirm the localization of phonons in Ba_10_Y_6_Ti_4_O_27_. Significant contributions to phonon scattering likely arise from the ordered, aperiodic structure through coherent features such as Y/Ti substitution at *t*=±0.1 (Figure 4 d,f) and the introduction of extra atomic layers with associated origin shifts at boundaries between Y/Ti and Ba crenels (Figure 4 b,g). In the three‐dimensional Debye–Callaway approach, these contributions are modelled as extended structural motif scattering, (as also observed in some quasicrystals[^44^, ^54^, ^55^]). This mechanism together with resonant scattering from the localized vibrations identified in the heat capacity dominates *κ*_latt_, with minimal contribution from point‐defect‐like disorder in Ba_10_Y_6_Ti_4_O_27_, reflecting the importance of the ordered aperiodic structure in controlling thermal transport. These two scattering processes produce glasslike temperature dependence, combining with the speed of sound to produce the low thermal conductivity. Such structural complexity is necessary to achieve *κ*_latt_<1 Wm^−1^ K^−1^ in oxide materials. Mo_18_O_52_, with comparably low thermal conductivity (Figure 5 a), has a complex crystal structure, featuring crystallographic shear planes and a range of Mo environments.^[31]^ The enhanced phonon scattering in Ba_10_Y_6_Ti_4_O_27_ reduces *κ*_latt_ over known Ba_6_Y_2_Ti_4_O_17_ (Figure 5 a) by a factor of two, associated with the aperiodic structure with complex bonding environments generating localized vibrations. Ba_10_Y_6_Ti_4_O_27_ introduces new structural and compositional motifs that can be used to design low *κ*_latt_ materials, and a distinct scaffold for optimization by substitution, defect control and processing. As an aperiodic titanate integrating tunnelling vibrational states, localised modes and extended structural motif phonon scattering, Ba_10_Y_6_Ti_4_O_27_ is based on structure and chemistry distinct from other best‐in‐class oxides (Figure 5 a), consistent with its classification as a new lead material.

## Conclusion

Ba_10_Y_6_Ti_4_O_27_ lies within an extensively studied phase field, emphasising the challenge that the size of chemical space poses for materials discovery. The quaternary was discovered with a workflow that guides synthesis through probe structure prediction to locate candidate regions of composition space for the isolation of new compounds. These regions are then prioritised by machine learning to identify those most likely to contain accessible compounds with low thermal conductivity. Although probe structure prediction and composition‐based machine learning do not deterministically predict the materials emerging from synthesis and their properties, these approximate models recognise unavoidable limitations in our ability to predict the synthesised structures and properties of complex materials with fine compositional resolution, and offer practical guidance for decisions made by the synthetic researcher. The resulting successive narrowing of compositional space first by formation energy then by physical properties of candidate regions, rather than individual compositions, focusses and thus enables the detailed experimental investigation needed to isolate and characterise metastable aperiodic Ba_10_Y_6_Ti_4_O_27_, where discontinuous occupancy modulation localises phonons to afford the targeted low *κ*. The unique structure and outperforming properties of Ba_10_Y_6_Ti_4_O_27_ highlight the need for effective routes to functional materials without structural precedent.

## Conflict of interest

The authors declare no conflict of interest.

## Supporting information

As a service to our authors and readers, this journal provides supporting information supplied by the authors. Such materials are peer reviewed and may be re‐organized for online delivery, but are not copy‐edited or typeset. Technical support issues arising from supporting information (other than missing files) should be addressed to the authors.

Supplementary
