# Supplementary material for: Discovery of a Low Thermal Conductivity Oxide Guided by Probe Structure Prediction and Machine Learning
Source: Angew Chem Int Ed Engl. 2021 Jun 17;60(30):16457–65. doi: 10.1002/anie.202102073 (PMC8362121; doi:10.1002/anie.202102073)
Supplement: Supplementary file 1 — Supplementary [file ANIE-60-16457-s001.pdf]

Angewandte Chemie
Eine Zeitschrift der Gesellschaft Deutscher Chemiker

Supporting Information

Discovery of a Low Thermal Conductivity Oxide Guided by Probe Structure Prediction and Machine Learning

*Christopher M. Collins, Luke M. Daniels, Quinn Gibson, Michael W. Gaultois, Michael Moran, Richard Feetham, Michael J. Pitcher, Matthew S. Dyer, Charlene Delacotte, Marco Zanella, Claire A. Murray, Gyorgyi Glodan, Olivier Pérez, Denis Pelloquin, Troy D. Manning, Jonathan Alaria, George R. Darling, John B. Claridge, and Matthew J. Rosseinsky**

anie_202102073_sm_miscellaneous_information.pdf

Table of Contents

Experimental Procedures 3
- Probe Structures 3
- Density Functional Theory (DFT) calculations 3
- Assembling the convex hull 3
- Stability of $Ba_{10}Y_6Ti_4O_{27}$ 3
- Phonon calculations 3
- Prediction of thermal conductivity using machine learning models 3
- Synthesis and processing 4
  - Isolation of $Ba_{10}Y_6Ti_4O_{27}$ 4
  - $Ba_{10}Y_6Ti_4O_{27}$ – 'hand ground' 4
  - $Ba_{10}Y_6Ti_4O_{27}$ – 'ball milled' 4
  - Processing to density 4
  - $Ba_6Y_2Ti_4O_{17}$ 4
- Powder Diffraction 5
- Transmission electron microscopy (TEM) 5
- TEM-EDX 5
- Precession electron diffraction tomography (PEDT) 5
- Selected area electron diffraction and high-angle annular dark field scanning transmission electron microscopy 5
- Scanning electron microscopy 5
- Thermal conductivity measurements 5
- Thermal expansions measurements 5
- Spectroscopy 6
- Diffuse reflectance 6

Results and Discussion 6
- Structure solution for $Ba_{10}Y_6Ti_4O_{27}$ 6
- Additional structural information 7
- Derivation of fractions of the structure represented by the different t values 7
- Creating the $10a_p$ pseudocubic supercell shown in Figure 2e 7
- Thermal expansion behaviour of $Ba_{10}Y_6Ti_4O_{27}$ and $Ba_6Y_2Ti_4O_{17}$ below 300 K 7
- Extraction of Grüneisen parameter and speed of sound 8
- Scanning electron microscopy 8
- Modelling the heat capacity of $Ba_{10}Y_6Ti_4O_{27}$ 8
- Modelling the thermal conductivity 8
- Phonon calculations and comparison against Raman data 9

Supporting Figures 10-25

Supporting Tables 25-28

References 28

Author Contributions 29

Experimental Procedures

Probe structures

A probe structure is a hypothetical structure with a unit cell large enough to sample the potential chemical bonding for a given composition, and therefore provide representative energies for the formation of single phases at that composition. In principle, any crystal structure prediction code (or combination of) could be used to generate probe structures, provided it can generate sufficiently complex structures for the given problem. For the systems addressed in this work, we concluded that codes developed in-house were well-suited to the systems studied.

Probe structures for the Y^{3+} – Ba^{2+} – Ti^{4+} – O^{2-} phase field were obtained as reported previously[14] using the Monte-Carlo – Extended Module Materials Assembly method (MC-EMMA), with 12 MC-EMMA runs performed for each composition and using the same module motifs. We assembled the phase field using a combination of known materials and probe structures (Table S1) yielding a total of 34 compositions in the phase field.

The probe structures for the Y^{3+} – Sr^{2+} – Ti^{4+} – O^{2-} phase field were used from our previous work[13] with energies computed using DFT (previously this was only done using force fields) generated by the Flexible Unit Structure Engine (FUSE, with three runs per composition), combined with known materials (Table S6). Probe structures and known compounds yielded a total of 30 compositions in the phase field.

Density Functional Theory (DFT) calculations

DFT calculations were performed using the Vienna Ab-initio Simulation Package (VASP)[S1], using the PBE[S2] functional and PAW[S3] potentials. Both the unit cell and atomic positions were relaxed until the forces were less than 0.01 eV Å^{-1}, with a plane wave cutoff energy of 520 eV. *k*-point grids were created to fulfil the minimum requirements of the following equation:

$$k_i \times r_i \geq 20$$

where k_i is the number of *k*-points along the direction *i* and r_i is the real space lattice vector along the same direction.

Assembling the convex hull

For each phase field, the final probe structure for each composition was computed by optimizing each of the structures from MC-EMMA / FUSE with DFT, taking the lowest energy structure as the probe structure. Duplicates were discarded along with structures from MC-EMMA runs that failed to converge. The combination of probe structures and known materials resulted in a total of 164 and 87 structures computed with DFT for the Y^{3+} – Ba^{2+} – Ti^{4+} – O^{2-} and Y^{3+} – Sr^{2+} – Ti^{4+} – O^{2-} phase fields respectively. The convex hull was then assembled using pymatgen[S4].

Stability of $Ba_{10}Y_6Ti_4O_{27}$

To assess the stability of $Ba_{10}Y_6Ti_4O_{27}$ we used the approximant model (4 × 1 × 1 supercell at t = 0) and performed a geometry optimization as described above for the DFT calculations, the energy above the convex hull was then assessed using the known materials in the probe structures section and pymatgen, which resulted in the approximant model being 0 meV atom^{-1} from the convex hull *i.e.,* it lies on the hull. These are 0 K calculations, and the observed requirement for a precise synthetic path to avoid decomposition between the synthetic and room temperature suggests that the material is subject to thermodynamic instability within this temperature range.

Phonon calculations

The phonons of the commensurate (*t* = 0) approximant model for $Ba_{10}Y_6Ti_4O_{27}$ were computed at the Γ point in a cell of composition $Ba_{20}Y_{12}Ti_8O_{54}$ using plane-wave based periodic DFT. VASP calculations were performed with the projector augmented wave approximation[S3] and the van der Waals corrected meta-GGA functional, SCAN+rVV10[S5] with a plane-wave cutoff energy of 550 eV. The cell parameters and atomic positions were relaxed until forces fell below 10^{-3} eV Å^{-1}, with a 3×5×2 *k*-point grid generated using a *k*-point spacing of 0.25 Å^{-1}. Normal mode frequencies and eigenvectors were then computed using the harmonic approximation where the Hessian was generated by finite differences with a step size of 0.015 Å. Of the 282 normal mode frequencies calculated, only the three rigid transformation modes had imaginary frequencies, all other frequencies were finite and positive. The elastic constant tensor was obtained from the normal mode calculation and used to calculate the bulk modulus using the Voigt–Reuss–Hill (VRH) approximation from the stress-strain relationship[S6].

Prediction of thermal conductivity using machine learning models

Machine learning models were trained on 1894 unique reported compositions and the associated experimental total and calculated lattice thermal conductivities available in the Gaultois et al.[16] and TEDesignLab[17] (accessed July 2019) thermoelectrics datasets. Models were constructed to treat these as separate distributions, but these did not lead to qualitatively different predictions nor model performance, so in the final models the two datasets are merged and are not differentiated.

Quaternary materials in the face and ternary materials along the edges of the phase field to be predicted were not included in the training data, but were included in the testing set to help assess performance of the final random forest and neural network models. Consequently, models were trained largely on materials that exist outside of this phase diagram, using a composition-based feature vector (described below) to predict compositions within the Y^{3+} – Ba^{2+} – Ti^{4+} – O^{2-} and Y^{3+} – Sr^{2+} – Ti^{4+} – O^{2-} phase fields.

Simple models using only chemical composition were first attempted, before incorporating more complex compositional features (Figure S10). Specifically, in terms of input features, approaches ranged from a one hot encoding of fractional elemental composition, to an enriched feature vector using the compositional featurizers available in Matminer[S7] and Magpie[S8]. Final models used the featurizer from Matminer (accessed August 2019), and the final feature vectors contained 121 features.

A range of different algorithms were used to build the models, ranging from simple linear regressors to more complex feed-forward neural networks; models built using neural networks and random forests outperformed other models, and produce qualitatively similar predictions (see Tables S2 and S3). All models were implemented using scikit-learn[S9], while neural networks were implemented in keras with the tensorflow backend[S10] in order to have greater control over the hyperparameters and training regimen. Adam was used as the optimizer[S11], with a default learning rate of 0.001; qualitatively no difference was observed between different optimizers nor optimizer hyperparameters. The final model used to construct Figure 1c and 1d was a 64 × 2 neural network, and the obtained values are the mean predictions of 5 instances.

Performance was evaluated using the mean squared error (MSE) and the coefficient of determination (R^2) of linear regressors when comparing the predicted thermal conductivity to the experimental values using both traditional, random k-fold cross validation (here k = 5), as well as leave one cluster out cross validation (LOCO CV). LOCO CV has been proposed by Meredig and co-workers as being more appropriate for determining the ability of ML models to predict material performance[S12], as it more accurately represents

the ability of a model to extrapolate between material families, and thus is useful to consider when trying to minimize the incidence of false positives. In addition to these statistical measures of performance, examples from the phase field of interest were included in the test set, and are shown visually in Figure S1, and numerically in Table S3. The final random forest and neural network models have a root mean squared error (RMSE) of 4.3 W m^{-1} K^{-1} across the entire dataset. The RMSE across the data subset below is 5.8 W m^{-1} K^{-1} for the final random forest models and 5.1 W m^{-1} K^{-1} for the final neural network models. When compared to the performance on the entire dataset, the similar RMSE in this data subset suggests the model is appropriate to use in this phase field, and the lower RMSE for the neural network models suggests they are more appropriate than the random forest models in this case and are thus used in this work to justify experimental prioritization (Figure 1).

The final neural network model estimates the expected thermal conductivities in the Y^{3+} – Ba^{2+} – Ti^{4+} – O^{2-} and Y^{3+} – Sr^{2+} – Ti^{4+} – O^{2-} phase fields (Figure S13). The range of values in the phase fields is similar, however there are larger areas of low thermal conductivity in the Ba system. Specifically, by the average thermal conductivity across the entire phase field is 13 ±6 W m^{-1} K^{-1} for the Ba system and 17 ±6 W m^{-1} K^{-1} for the Sr system. In the lower right composition region where χ_{BaO} or χ_{SrO} > 0.5, the difference is larger, with an average thermal conductivity of 6 ±2 W m^{-1} K^{-1} for the Ba system and 13 ±3 W m^{-1} K^{-1} for the Sr system. Following the use of probe structure calculations to identify compositional regions where new materials can be experimentally isolated, the average predicted thermal conductivity of these regions was used to prioritize the compositional region with lower predicted thermal conductivity where the new material $Ba_{10}Y_6Ti_4O_{27}$ was found. (Figure 1).

When using ML predictions of performance to screen material candidates, there are considerable resources invested when embarking on an experimental campaign. Consequently, it is important to consider the likelihood of a model predicting false positives, which in this case, means predicting a low thermal conductivity when a material has a high thermal conductivity. In the screening and prioritization context of this work, it is of utmost importance to minimize the incidence of false positives (materials with high κ but predicted to be low), even at the expense of overall model accuracy. Furthermore, in addition to comparing lines showing the 1:1 correspondence of a perfect model, we also examine lines that lie below 95% and 99% of all predictions (Figure S10). Additionally, model performance was examined as a function of the dataset size, and the loss over epoch was assessed where appropriate to check for overfitting (Figure S11).

Synthesis and processing:

Isolation of $Ba_{10}Y_6Ti_4O_{27}$

To isolate $Ba_{10}Y_6Ti_4O_{27}$, a total of 32 compositions were sampled (Figure S2a-c), with the search starting from the two compositions: $Ba_{0.667}Y_{0.167}Ti_{0.167}O_{1.25}$ and $Ba_{0.5}Y_{0.333}Ti_{0.167}O_{1.33}$ which both lie within the compositional target region identified as most likely to contain new phases with low thermal conductivity (Figure 1b and d). From the two initial compositions, $Ba_{0.5}Y_{0.333}Ti_{0.167}O_{1.33}$ contained fewer reflections from known phases (primarily Ba_2TiO_4 and $Ba_3Y_4O_9$) and more prominent reflections arising from an unknown phase, and so was the basis for EDX study (Figure S2d), which identified the presence of a new phase. The remaining 30 compositions were sampled guided by the EDX measurement of the composition of the new phase until we obtained high purity materials (*i.e.,* no additional phases could be detected in standard laboratory-PXRD experiments), which was achieved for samples at the nominal composition $Ba_{0.53}Y_{0.288}Ti_{0.178}O_{1.32}$. All the exploratory syntheses and powders of the new quaternary phase prepared for crystallographic measurements were synthesized through hand grinding as described below.

$Ba_{10}Y_6Ti_4O_{27}$ – 'hand ground'

During initial synthesis trials, variations in the synthesis temperature were examined, 1300 °C – 1450 °C in steps of 50 °C and annealed for 48 hours, under these conditions the new phase could be formed over the temperature range 1300 °C – 1400 °C. If the material was heated further (or at higher temperatures such as 1450 °C) then decomposition was observed, yielding the formation of known phases such as $Ba_6Y_2Ti_4O_{17}$ and Ba_2TiO_4 similar to results seen in Figure S3 Using this refined synthesis temperature, exploratory synthesis was then performed at the 30 compositions (Figure S1), by heating samples at 1400 °C for 48 hours in a tube furnace under flowing synthetic air, with a heating and cooling rate of 5 °C min^{-1}; annealing samples for longer times at this temperature results in sample decomposition.

For starting materials TiO_2 (99.8% purity, 99.995% used in structural characterization sample, Alfa Aesar), Y_2O_3 (99.99% purity, 99.999% used in structural characterization sample, Alfa Aesar) and $BaCO_3$ (99.997% purity, Alfa Aesar) were used. The starting materials were weighed out in stoichiometric quantities and hand ground in small amounts of acetone (less than 5 cm^3), allowed to dry in lab air prior to heating. All samples were synthesized on a 200 mg scale, with starting materials dried prior to weighing; TiO_2 and $BaCO_3$ dried at 200 °C and Y_2O_3 dried at 900 °C for a minimum of 6 hours. The sample used for all of the structural characterization was synthesized via this route and scale, at the nominal composition $Ba_{0.53}Y_{0.29}Ti_{0.18}O_{1.32}$.

$Ba_{10}Y_6Ti_4O_{27}$ – 'ball milled'

Samples for physical property measurements were synthesized as follows at the nominal composition $Ba_{0.53}Y_{0.29}Ti_{0.18}O_{1.32}$: Starting materials were dried and weighed out as above for a 5 g scale. The combined starting materials were then milled in a Pulverisette7 classic line ball mill, with seven 10 mm ZrO_2 balls in 10 cm^3 of ethanol for 54 cycles of 15 minutes at 350 rpm with the direction reversed between cycles with a ten-minute pause. After milling, the starting materials were dried on a hot plate at 35 °C with stirring, once dried the material was ground by hand, and stored in a desiccator until used. The starting material was divided into 0.5 g aliquots which were spread as loose powder in alumina boats (dimensions 10 × 40 mm in size). With two aliquots to a furnace, samples were then placed under dried synthetic air at room temperature for 2 hours, then heated at 5 °C min^{-1} to 675 °C, dwelling for 2 hours, heated to 1350 °C for 2 hours before cooling back to room temperature at 5 °C min^{-1}. The resulting powder was then X-rayed and stored in a desiccator until processed for physical property measurements. We additionally tested samples milled as above, but for only 4 cycles and synthesis times of 4, 8, 12, 18 and 24 hours. All of the additional conditions tested beyond the above optimized protocol resulted in partial decomposition of the new quaternary phase (similar to results shown in Figure S3). This decomposition over time at the temperature required for synthesis, also observed in the hand ground samples, demonstrates the need for the exact synthesis protocol defined above to isolate the material, and is consistent with metastability.

Processing to density

The metastable nature of the new quaternary material also created difficulties for densification, which required specific protocols as processing at high temperatures resulted in decomposition of the phase (Figure S3b). To obtain phase pure, dense pellets of the new quaternary, samples were prepared under vacuum (< 5 × 10^{-2} mbar) via spark plasma sintering (SPS) in a tungsten carbide die set using a commercial Thermal Technology LLC DCS10 furnace. For each pellet, 0.7 g of powder was used (from combining aliquots from the synthesis above). 800 MPa of uniaxial pressure was applied on powder using a 10 mm inner diameter tungsten carbide die set (WC with 6% Co binder) lined with carbon foil. The temperature was monitored through a bore-hole in the side of the

die set using a pyrometer, and the emissivity of the die set was not accounted for, so the true temperature of the powder during processing is expected to differ from the measured temperature. Samples were then heated to 320 °C at a rate of 50 °C min^{-1}, annealed for 20 seconds, before cooling back to room temperature at the same rate and the pressure released at a rate of 200 MPa min^{-1}. Samples dwelled for five minutes before the pressing sequence was repeated, once the samples had returned to room temperature and the pressure was released the sample was removed. During the heating steps, the temperature of the sample would typically overshoot by 20 °C before returning to the target temperature. The SPS procedure resulted in pellets with a density of 5.16 g cm^{-3}, which is 96.6% of the density of the commensurate approximant model (5.34 g cm^{-3}).

$Ba_6Y_2Ti_4O_{17}$

Samples of $Ba_6Y_2Ti_4O_{17}$ were synthesized as reported previously[12]. Pellets of $Ba_6Y_2Ti_4O_{17}$ were processed using the same SPS furnace as above under dynamic vacuum (<5 × 10^{-2} mbar) using 1.0 g of powder. 50 MPa of uniaxial pressure was applied over a 10 mm internal diameter graphite (ISOCARB85) die. Samples were heated to 1480 °C at a rate of 300 °C min^{-1}, followed by heating to 1500 °C at 100 °C min^{-1}, with a 20 second dwell, before power to the furnace was shut off and the sample allowed to cool rapidly to ambient conditions. Measurements of thermal conductivity were performed on $Ba_6Y_2Ti_4O_{17}$ pellets which were 93.3% dense compared against the crystallographic model.

Powder Diffraction

For the initial phase diagram exploration and for screening of samples produced for physical properties measurements PXRD patterns were measured on a Bruker D8 Advance diffractometer using a monochromatic CuKα_1 source (λ = 1.54056 Å) in transmission foil geometry.

The PXRD pattern used in the initial structure solution was collected on a Phillips PANalytical diffractometer with a monochromatic CoKα_1 source (λ = 1.78901 Å) in Bragg-Brentano reflection geometry. For the final structural refinement, PXRD data were collected at the Diamond Light Source on the I11 beamline (λ = 0.82526 Å). The time of flight Neutron Powder Diffraction (NPD) data used as a part of the structure solution was collected at the POLARIS beamline at the ISIS neutron source on ~ 150mg of material contained in a capillary.

Transmission electron microscopy (TEM)

For TEM measurements, a small quantity of powder was dispersed in ethanol and ground in an agate mortar. A drop of the suspension was then deposited and dried on a copper grid with an amorphous holey carbon film.

TEM-EDX

For the initial sample from the exploratory synthesis at the composition $Ba_{0.5}Y_{0.333}Ti_{0.167}O_{1.33}$ synthesized by the hand ground route, Energy Dispersive X-ray spectroscopy (EDX) was performed with a JEOL 2000FX equipped with an EDAX detector on 10 particles (data in Figure S2d). Correction factors, for the different elements, were estimated measuring the EDX spectra of appropriate standards. Standards purity was confirmed using X-ray diffraction. Quantification was performed using the EDAX Genesis software. Only Y, Ba, Ti and O were observed in the samples.

To compliment the structural studies and TEM imaging, we collected EDX data on the same sample using a 200 kV JEOL 2000FXII microscope. EDX spectra were collected on 30 crystals for several minutes in order to obtain a suitable signal-to-noise ratio (data in Figure 2a). The quantification data, for each element, were corrected using a correction factor determined from a standard.

Precession Electron Diffraction Tomography (PEDT)

PEDT experiments were performed on a JEOL 2010 microscope operating at 200 kV and equipped with a Nanomegas DigiStar precession module. PEDT data sets were recorded on several different crystals. For all data sets the precession angle was set to 1.2° and goniometer tilt steps of 1° were performed over a range of 100°, with a non-oriented diffraction pattern recorded at each step. PEDT data were then processed using PETS[S13] and Jana2006[S14, S15] programs.

Selected-Area Electron Diffraction (SAED) and High-angle annular dark field scanning transmission electron microscopy (HAADF-STEM)

The SAED patterns were collected using a Schottky field emission gun equipped JEOL JEM 2100FCs microscope operating at 200 kV. HAADF images were also recorded using this microscope in STEM mode. The material turned out to be extremely beam sensitive when using STEM illumination conditions. It was not possible to image using typical conditions. The approach taken was to focus on a region adjacent to the area of interest and then move the specimen slightly, to an undamaged region. This led to noisy slightly out of focus images, which were however suitable to determine the structural and compositional details discussed in the text.

Scanning electron microscopy

Scanning Electron Microscopy (SEM) was performed using a Tescan S8000. Prior to imaging pellets were coated with a thin layer of carbon with a Quorum sputter coater in order to avoid charging.

Thermal conductivity measurements

Thermal diffusivity data (α) were measured through the laser flash method using a Netzsch LFA 457. The pellets (approximately 10 mm in diameter and 1.5-2 mm thick) were coated in colloidal graphitic carbon before being placed inside the sample chamber, which was evacuated and purged three times with synthetic air before measurement. Measurements were taken under a flow (100 mL min^{-1}) of synthetic air. Data were collected in 25 K steps in the temperature range 298 to 573 K using a heating rate of 3 K min^{-1} and five-minute equilibration at each temperature before measurement. Thermal diffusivities were obtained by fitting the Cowan model to raw data (Figure S14 and S15). Four measurements were performed at each temperature and their diffusivities were averaged, with the standard deviation of points being below 0.6 %. Sufficient time (180 seconds) separated each of the four measurements allowing for equilibration from local heating of the sample from the laser. Heat flux profiles were measured using a Netzsch DSC 404 F1 differential scanning calorimeter under a 50 mL min^{-1} flow of synthetic air. Continuous data were recorded from 323 to 573 K using a heating rate of 10 K min^{-1}. Data were measured from a sapphire standard of similar mass (25 mg) under identical conditions to determine the heat capacity (C_P) of the $Ba_{10}Y_6Ti_4O_{27}$ and $Ba_6Y_2Ti_4O_{17}$ samples. Errors on the heat capacity are assumed to be 5% as advised by the manufacturer. The thermal conductivity (κ) was calculated by combining the diffusivity and heat capacity data through $\kappa(T) = \alpha(T)\cdot C_P(T)\cdot\rho$, assuming a constant pellet density ρ which was determined using the Archimedes method.

Low temperature heat capacity data were measured on a Quantum Design Physical Properties Measurement System (QD PPMS) heat capacity option, with the relaxation method. The sample was affixed to the stage using N-grease, the contribution of which to the heat capacity was subtracted by measuring an addendum prior to sample measurement. Three data points were collected at every temperature to measure an average.

Low temperature thermal conductivity data were measured using a two-contact pill geometry on a QD PPMS using the Thermal Transport Option. A pellet of $Ba_{10}Y_6Ti_4O_{27}$ 10 mm in diameter sputtered with gold on both sides, followed by using two

component silver epoxy to fix the copper contacts onto each face of the sample. The low temperature data is shown up to 150 K due to the error in accounting for the radiative correction becoming large at higher temperatures. The data were also measured using a 4 contact bar geometry which showed, within error, the same results.

Thermal expansion measurements

Variable-temperature powder synchrotron X-ray diffraction data were collected on $Ba_{10}Y_6Ti_4O_{27}$ using beamline I11 (λ = 0.825058 Å) at Diamond Light Source, U. K., utilizing two different configurations. Diffraction patterns were measured in 20 K steps from 80 – 300 K using the Oxford Cryostream Plus stage with sample loaded in 0.2 mm diameter borosilicate capillaries. The ramp rate was 6 K min^{-1}, and the sample temperature allowed to equilibrate for 10 min prior to measurement. Data in the temperature range 12 – 80 K were collected in 20 K steps using a closed helium cycle PheniX cryostat (Oxford Cryosystems, U.K.) from a thin coating of $Ba_{10}Y_6Ti_4O_{27}$ powder on a piece of aluminium wire mounted using an adapted sample holder[S16]. The ramp rate was 6 K min^{-1}, and again the sample was allowed to equilibrate for 20 min prior to each measurement.

Variable-temperature powder X-ray diffraction data were collected on $Ba_6Y_2Ti_4O_{17}$ using a Rigaku SmartLab instrument equipped with 9 kW rotating anode source providing a parallel beam of Mo $K\alpha_1$ (λ = 0.73900 Å) radiation. The sample was measured in reflection geometry on an OxfordCryosystems PheniX closed cycle helium cryostat stage. Data were recorded over 5 – 50° 2θ from 300 – 12 K in steps of 20 K with a sixty-minute equilibration at each temperature before measurement. Lattice parameters were extracted through Rietveld refinement.

Spectroscopy

Raman spectra were measured on polycrystalline samples of $Ba_{10}Y_6Ti_4O_{27}$, $BaCO_3$ and $Ba(OH)_2$ in a Renishaw inVia Raman Spectrometer equipped with a laser of excitation wavelength 633 nm.

Diffuse Reflectance

UV-visible spectra were recorded from $Ba_{10}Y_6Ti_4O_{27}$ powder on an Agilent Cary 5000 UV-vis_NIR spectrophotometer in the wavelength range 175-800 nm. The Kubelka-Munk function was obtained for each sample using the reflectance R in the equation $F(R) = (1-R)^2/2R$. The Tauc method was then used to determine the band gap of $Ba_{10}Y_6Ti_4O_{27}$ by plotting $(h\nu F(R))^2$ against energy (Figure S8b).

Results and Discussion

Structure solution for $Ba_{10}Y_6Ti_4O_{27}$

The complexity of the powder diffraction pattern of $Ba_{10}Y_6Ti_4O_{27}$ led to the use of precession electron diffraction to identify the unit cell of the new composition (after attempts to identify the unit cell from PXRD failed). Electron diffraction defined an aperiodic monoclinic cell (3+1)D with the parameters: a = 6.280(2) Å b = 6.077(2) Å c = 10.462(3) Å β = 94.76(3)°, with modulation vector, **q** = 0.2498(7)a* - 0.0013(12)c* in the super space group P2/m(g0a)0s, with the origin at the fractional co-ordinate (0,1/4,0,0). These results identified the modulation (Figure 3), with the indexation confirmed by Le Bail fitting against laboratory PXRD data (Figure S16).

Initial attempts to solve the aperiodic structure directly were unsuccessful due to the high relative intensities of the satellite reflections, however as $\mathbf{q} \approx \mathbf{q}_{comm}$ = 0.25a* - 0c* from electron diffraction, it was pragmatic to first solve a commensurate approximation of the structure using $\mathbf{q}_{comm}$, then to progress from this to solve the full modulated structure. The aperiodic cell was approximated to the commensurate cell 10.46 × 6.08 × 24.8 Å with the angles 90 × 94.8 × 90° in the space group P2/c, derived from the superspace group in Jana2006[S14, S15] based upon a 4 × 1 × 1 supercell with the origin in x_4 at 0. Note when deriving the commensurate approximant unit cell, at t = 0 for a 4 × 1 × 1 supercell the symmetry is P2/a, which is a non-standard setting of P2/c, Jana2006 then performs the transformation to the standard setting by the following matrix which results in transposition of the a and c axes:

$$\begin{bmatrix} 0 & 0 & 1 \\ 0 & -1 & 0 \\ 1 & 0 & 0 \end{bmatrix}$$

The initial structure solution for the commensurate model was solved using laboratory PXRD (λ = monochromatic $CoK\alpha_1$ = 1.78901 Å) collected on a Phillips PANalytical diffractometer over the d-space range 20 – 0.99 Å. The location of “heavy atoms” (Ba, Y and Ti) was determined using Superflip[S17, S18] as implemented in Jana2006, with a Le Bail fit performed to determine an initial unit cell and profile parameters (Figure S16). To determine the species present on the cation sites, each site was initially given an occupancy factor of 1 Ba^{2+}. A Rietveld refinement was then performed, refining only the overall scale parameter and the occupancy factors of all but one cation site, which was fixed at 1 Ba^{2+}. Species were then assigned based upon the refined occupancy parameters based upon the assumption that $Y^{3+} \approx ⅔ \times Ba^{2+}$ and $Ti^{4+} \approx ⅓ \times Ba^{2+}$ in terms of electron density. This resulted in the location of 11 unique cation sites: 6 × Ba, 3 × Y and 2 × Ti. Fourier difference maps were calculated to locate possible O sites, skipping peaks closer than 1 Å from any existing site. Fourier mapping lead to the identification of 14 unique O sites (yielding a total of 25 sites in the structure) within the unit cell, the structure solution contained the composition $Ba_{10}Y_6Ti_4O_{27}$ or $Ba_{0.5}Y_{0.3}Ti_{0.2}O_{1.35}$, within 1 e.s.d. of the EDX measured composition.

A Rietveld refinement was then performed for the whole commensurate structure (Figure S4a), with the resulting lattice parameters a = 10.42982(10) Å, b = 6.10124(6) Å, c = 24.8216(2) Å, β = 94.8329(6)° with χ^2 = 3.54 with 73 structural parameters.

The fractional co-ordinates from the commensurate approximate solution were used as the basis to solve the full modulated structure. To construct a starting model, we folded the commensurate structure back onto the aperiodic sub-cell by dividing all of the co-ordinates along the c-axis by four (as the commensurate approximant is ~ 4 fold supercell along c) the x and z coordinates were then switched for each of the sites to take account of the different unit cell settings between the commensurate and full aperiodic unit cells.

The aperiodic structure was solved using a combination of Time-of-Flight (TOF) Neutron Powder Diffraction (NPD) over the d-space range 17.85 – 0.81 Å and the previous PXRD data. The modulation vector and unit cell from the electron diffraction and the atomic co-ordinates from the commensurate solution were used as the starting point for the solution, all of which were initially fixed. Modulations for the occupancy of all atoms were determined by sequentially generating random modulation waves in Jana2006, a process by which random amplitudes are applied to each site in the structure and then refined with the Rietveld method. Once converged, the modulation waves are re-randomized and refined. This process was repeated until no lower goodness of fit could be located (~ 50 repeats).

For all 25 sites in the structure (11 cation and 14 anion), the occupational modulation waves were converted into discontinuous crenel functions[S19] – a block wave where the occupancy of a site can be 1 or 0 along x_4 (with an example function plotted in Figure S5

for the Y2 site). Modelling the occupancies with discontinuous crenels greatly improved the fit over continuous modulation waves[23]. Given that the atomic occupancies are all discontinuous, the modulated (3+1)D description is equivalent to a 4D quasicrystal structure by a change in choice of axes; here we use the aperiodic description. The crenel function is refined through two parameters, Δ and $x4_0$. Δ indicates the width of the part of the crenel where the occupancy is equal to 1 in t (the internal co-ordinate along x_4) and $x4_0$ defines the centre point of the region of the crenel where the site occupancy is equal to 1 in t. The approximant (*i.e.,* commensurate model) corresponds to all values of Δ = 0.25 (as a result of the symmetry of the superspace group) and **q** = $\mathbf{q}_{\mathbf{comm}}$ = 0.25a*. The occupancy modulation waves were converted to crenel functions by determining the $x4_0$ values which correspond to the center of peaks in the occupancy modulation waves and all Δ values fixed to be equal to 0.25, resulting in the stoichiometric composition $Ba_{10}Y_6Ti_4O_{27}$. The crenel functions for all of the cation sites are plotted in Figure S6a and a subset of cation sites in Figure 4a (all of the Y and Ba sites within the internal co-ordinate range x = 0 – 1, y = 0 – 1, z = 0 – 1/3 and t = 0 – 1).

An initial structural refinement was then created by refining the unit cell parameters, modulation vector, atomic co-ordinates and crenel functions against the combined NPD and laboratory PXRD (Figure S17), with a converged global χ^2 value of 2.18 for 110 structural parameters. We note that, contrary to most aperiodic structure solutions, this model increases the number of structure parameters, because it is significantly more complicated than the commensurate approximant. For example, the approximant model does not include any of the Y – Ti ordering on the Y2 site. For comparison the modulated structure model was also refined solely against the PXRD data, where χ^2 = 2.62, compared to 3.54 for the commensurate model (Figure S4).

When this model is plotted in real space, it is observed that a significant proportion of the structure has a low density, resulting in the overall density of the model falling to 5.0(10) from 5.32(9) g cm^{-3} from the approximant model, when compared with the experimental observation of 5.2 g cm^{-3}. It was concluded that the satellite reflections were not suitably resolved / collected in the NPD and laboratory PXRD data to refine a complete model.

To refine the final model, high resolution synchrotron PXRD (S-PXRD λ = 0.82526 Å, over the d-space range 18 – 0.52 Å, step size = 0.008°) was collected at the I11 beamline at the Diamond Light Source. Refinement against S-PXRD data resulted in a converged model with a higher total density of 5.30(7) g cm^{-3}, with a χ^2 equal to 91.4 for 120 structural parameters. The value of χ^2 results from the diffraction pattern being over-collected in order to observe as many satellite reflections as possible. We obtain a reasonable R_{wp} of 8.42% (difference between observed and calculated data points). Due to the over collection of the data, the R_{exp} (the expected fit parameter) is extremely low (0.88 %), and as $\chi = R_{wp} / R_{exp}$ we therefore have a large value of χ [S20].

In the final model, three of the eleven cation sites were split into two components (Ba5, Y1 and Y2). Each component is described by a crenel function. The first (major) component is modelled as every other site detailed above (noted as MX where M is the species and X is the site number of that species). For the second (minor) component of each site (denoted with a *′ i.e.,* MX′), the average x, y and z positions and thermal parameters were constrained to be equal to the first part. Δ was constrained to fulfil Eq. S1 such that the total Δ is constrained to equal 0.25:

$$\Delta_{M'} = 0.25 - \Delta_M \quad \text{(S1)}$$

The Ba5′ and Y1′ sites ($\Delta_{M'}$ = 0.0625(2) and 0.0540(3) respectively) include a positional modulation, modelled with sine and cosine waves, orthogonalized over the width of the crenel function, which displaces their position away from the average. $x4_0$ was then set to fulfil the Eq. S2:

$$x4_{0M'} = x4_{0M} + \tfrac{1}{2}\Delta_M + \tfrac{1}{2}\Delta_{M'} \quad \text{(S2)}$$

where M is the first part of the site, M′ is the second part of the split site and Δ is the width of the crenel. These two restrictions create a crenel function with a fixed total width of 0.25 and are located such that they continue from each other in t (Figure S5).

For the Y2 site, the Y2′ part of the crenel was set such that Y swaps to being occupied by Ti ($\Delta_{M'}$ = 0.058(6)). The partial substitution of the Y2 site then allows the model to contain the Y swapped for Ti in the structure at t = 0.1 and t = – 0.1 as confirmed in TEM images in Figure 4e-4g and results in the non-stoichiometry of the refined model ($Ba_{10}Y_{5.54(16)}Ti_{4.46(16)}O_{27}$). Powder Crystallographic Information Files (CIFs) are included in the supplementary materials for the approximant and final aperiodic solutions refined against the I11 data, all of the powder diffraction data used in the various stages of the structure solution are available as part of the data repository associated with this work.

Additional structural information

As **q** = 0.24964(2) a* - 0.00021(4) c* ~ (¼-δ) a*, the superspace model predicts that as we continue along external space, we also move slowly along t such that the effective origin of the next supercell will not be t, but t - 4δ. As δ ~ 0.0002, the origin will shift by ¼ in t after ~ 1250 repeats of the supercell, as in the section in Figure 4b, where the associated shift in the cation columns from Figure 4c is apparent. Their presence in the HAADF STEM images in Figure 4g is further evidence of the aperiodic nature of the material. Given the very limited extent of these features we cannot reproduce their exact atomic structure in the model, where they appear more diffuse, though this may represent some disorder in their location. These features clearly contribute very little to the overall scattering in the diffraction data.

Note that in the full structure, the superspace symmetry inverts the crenel function in t for half the Y2 sites. The inversion of the Y2 sites leads to a second titanium-rich region as in the section t = -0.1 (Figure S6) which replaces the other half of the Y2 sites not replaced in Figure 4d, corresponding to approximately 20% of the structure. This, combined with the titanium rich region around t = 0.1 (also corresponding to ~ 20 % of the structure, blue shaded Figure S6), leaves approximately 60% of the structure with no Y2 for Ti substitution, as shown for t = 0, in Figure S6d.

Derivation of fractions of the structure represented by the different t values

The estimate of the percentage of the structure described by the 4 x 1 x 1 supercell at t = 0 was derived based upon the relative widths of two parts of the crenel function on the Y2 site. Using the assumption that where the Y2 site is occupied by Y we have the t = 0 approximant structure and where Y2 is occupied by Ti we have either the structure described at t = -0.1 or 0.1 (Figure S6), it is possible to approximate the fraction of the structure that is represented by the model at t = 0.

The minor component of the Y2 site has Δ = 0.058(6) occupied by Ti, which as a fraction of the total width of the crenel is 0.058(6) ÷ 0.25 = 0.23(2), or 23(2)%. As the superspace symmetry inverts the crenel of half of the Y2 sites, only half of the Y2 sites are substituted at any one time (Figure S5), therefore doubling the proportion of the structure which contains Y2 sites substituted with Ti to 46(4) %. It follows, that the regions of the crenel which do not contain any Y2 sites substituted for Ti are therefore represented by the structure at t = 0, which equates to 54(4)% of the structure. With the magnitude of the e.s.d. on the refined crenels, and as the Y:Ti ratio approximates to 80:20, we approximate that the ratio of the two components of the structure is ~ 60:40.

Creating the 10a$_p$ pseudocubic supercell shown in Figure 2e

The 10a$_p$ pseudocubic supercell was created using the lattice transformation: **a**′ = **c**+2**a**-5**b**, **b**′ = **c**+2**a**+5**b** and **c**′ = **c**-3**a**, using the a, b and c lattice parameters from the commensurate approximant unit cell described above (*a* = 10.42982(10) *b* = 6.10124(6) *c* = 24.8216(2) Å, α = 90, β = 94.8329(6) and γ = 90 °) yielding a pseudo-cubic supercell with the parameters *a*′ = *b*′ = 43.525, *c*′ = 41.539 Å, α′ = β′ = 90.467, γ′ = 88.991°.

Thermal expansion behaviour of $Ba_{10}Y_6Ti_4O_{27}$ and $Ba_6Y_2Ti_4O_{17}$ below 300 K

Variable-temperature X-ray powder diffraction in the temperature range of 12 – 300 K was performed to extract the volumetric thermal expansion coefficient (α_V) of both the new $Ba_{10}Y_6Ti_4O_{27}$ and the known $Ba_6Y_2Ti_4O_{17}$ quaternaries. The unit cell volumes of $Ba_{10}Y_6Ti_4O_{27}$ and $Ba_6Y_2Ti_4O_{17}$ obtained from Rietveld refinement are plotted as a function of temperature in Figure 5d and Figure S20, respectively. The unit cell volume of $Ba_{10}Y_6Ti_4O_{27}$ remains constant from 12 to 20 K, and then increases from 40 K. Between 120 – 300 K, a monotonic increase in unit cell volume is observed for $Ba_{10}Y_6Ti_4O_{27}$. The unit cell volume of $Ba_6Y_2Ti_4O_{17}$ as a function of temperature behaves in a similar way and expands from 12 K up to 300 K, but presents a less-pronounced plateau below 20 K.

These non-linear variations in unit cell volume as a function of temperature for both materials are described through the polynomial equation $V_0 + V_1T + V_2T^2 + V_3T^3 + V_4T^4$ by the terms in Table S4.

These differences in behaviour between $Ba_{10}Y_6Ti_4O_{27}$ and $Ba_6Y_2Ti_4O_{17}$ are reflected in their volumetric thermal expansion coefficients (α_V) which are plotted Figure S21. The volumetric thermal expansion coefficients were calculated as $\alpha_V = 1/V_{300\,K}\cdot(\Delta V/\Delta T)$, with units of K^{-1}, where $V_{300\,K}$ is the unit cell volume of the materials at 300 K. At the lowest temperatures (12-20 K), $Ba_{10}Y_6Ti_4O_{27}$ exhibits a negative thermal expansion coefficient which becomes positive at ≥40 K before increasing to 300 K. $Ba_6Y_2Ti_4O_{17}$ exhibits a linear volumetric thermal expansion coefficient, increasing monotonically from 12 to 300 K, in stark contrast to the non-linearity observed for $Ba_{10}Y_6Ti_4O_{27}$, which indicates significant changes in vibrational frequencies, and therefore interatomic bonding, as a function of temperature.

Extraction of Grüneisen parameter and speed of sound

The Grüneisen parameter (γ) has several formulations (Eq. S3) combining the volumetric thermal expansion coefficient (α_V), heat capacity (C_P), molar volume (V_m), bulk modulus in GPa (B_T) which is estimated from the elastic constants using DFT, compressibility (β), and density (ρ)[S21, S22]:

$$\gamma = \frac{\alpha_V B_T V_m}{C_V} = \frac{\alpha_V V_m}{C_V \beta} = \frac{\alpha_V B_T}{C_V \rho} \qquad \text{(S3)}$$

which were used to assess the Grüneisen parameters of $Ba_{10}Y_6Ti_4O_{27}$ and $Ba_6Y_2Ti_4O_{17}$ over the temperature range 12 – 300 K.

The specific heat at constant volume (C_V) can be determined from the specific heat at constant pressure (C_P). At low temperatures $C_P \approx C_V$, but at high temperatures, C_V can be determined from Eq. S4:

$$C_V = C_P - \alpha_V^2 B_T V_m T \qquad \text{(S4)}$$

The compressibility is the inverse of the bulk modulus, and through the thermodynamic relationship $v_s^2 = 1/\rho\beta$ [S23], the velocity of sound (v_s) can be extracted from the Grüneisen parameter through Eq. S5:

$$v_s = \sqrt{\frac{\gamma\, C_P}{\alpha_V}} \qquad \text{(S5)}$$

At low temperatures (<40 K), γ follows the thermal expansion coefficient of $Ba_{10}Y_6Ti_4O_{27}$ and becomes increasingly negative with decreasing temperature (Figure 5d). In contrast, the Grüneisen parameter of $Ba_6Y_2Ti_4O_{17}$ is positive at all temperatures (Figure S22). The extracted Grüneisen parameters of $Ba_{10}Y_6Ti_4O_{27}$ and $Ba_6Y_2Ti_4O_{17}$ are given in Tables S7 and S8, respectively.

Scanning electron microscopy

The porosity of the processed ceramics was assessed using scanning electron microscopy (SEM) as the porosity can significantly affect the measurement of thermal conductivity from an object. The images (Figure S23) show the processed $Ba_{10}Y_6Ti_4O_{27}$ ceramics to have minimal porosity, in agreement with the measured densities of the objects.

Modelling the heat capacity of $Ba_{10}Y_6Ti_4O_{27}$

The heat capacity of $Ba_{10}Y_6Ti_4O_{27}$ was modelled using a combination of Debye and Einstein functions, with the expressions shown in Eq. S6 and S7 respectively [S24]:

$$C_V = 9N_{Di}R\left(\frac{T}{\theta_{Di}}\right)^3 \int_0^{\theta_{Di}/T} \frac{x^4 e^x}{(e^x-1)^2}\,dx \qquad \text{(S6)}$$

where $x = \hbar\omega/k_BT$, N_{Di} is the number of atoms per formula unit with Debye temperature θ_{Di}, and R is the molar gas constant.

$$C_V = 3N_{Ei}R\left(\frac{\theta_{Ei}}{T}\right)^2 \frac{e^{\frac{\theta_{Ei}}{T}}}{\left(e^{\frac{\theta_{Ei}}{T}}-1\right)^2} \qquad \text{(S7)}$$

where N_{Ei} is the number of atoms per formula unit with Einstein temperature θ_{Ei}. The Einstein contribution represents that of localized oscillators with a singular vibrational frequency, and thus represents non-propagating modes, in contrast to the propagating phonons of the Debye model.

It was necessary to use a linear contribution to fit the heat capacity of $Ba_{10}Y_6Ti_4O_{27}$. Usually, these terms are attributed to electronic contribution to the heat capacity, but can also arise from any two-level quantum system wherein an atom can tunnel between two states, and is commonplace in disordered solids such as glasses and relaxor ferroelectrics [S25, S26]. In $Ba_{10}Y_6Ti_4O_{27}$, this term is likely to then be due to the incommensurate modulation of the structure where modulations will likely create atomic positions that are close in energy. In intermetallic quasicrystalline materials, an electronic component exists, making a possible lattice contribution to the linear term difficult to deconvolute[49]. As such, this is the first observation of a linear term in the heat capacity (Figure 5c) of a proposed quasicrystal that arises from vibrational tunnelling states. The fit of the model to experimental data, along with extracted parameters are shown in Figure S7 and Table S9. Two Debye and Einstein terms are needed to accurately model the heat capacity at high and low temperatures, respectively. The need for multiple Debye terms can arise from dissimilar masses or bonding within the lattice and this approach has been used previously to model the heat capacity of oxides[S27, S28]. The two Debye temperatures 290(2) K (θ_{D1}) and

800(10) K (θ_{D2}) are associated with the low frequency and high frequency modes, respectively. The two Einstein temperatures 95.5(5) K (θ_{E1}) and 54.3(4) K (θ_{E2}) are associated with localized vibrational modes at a single frequency.

Modelling the Thermal Conductivity

The thermal conductivity was modelled using the integral form of the thermal conductivity

$$\kappa_{latt} = \frac{v_s}{3}\int_0^{\omega_D} C(\omega)l_{ph}(\omega)d\omega \quad \text{(S8)}$$

where v_s is the average speed of sound as extracted from the Grüneisen parameter, ω_D is the Debye frequency extracted from the heat capacity, $C(\omega)$ is the frequency dependent heat capacity, and $l_{ph}(\omega)$ is the frequency-dependent phonon mean free path.

The phonon mean free path was calculated by summing different frequency-dependent components as follows[52]:

$$l_{ph}(\omega) = (l_{pd}^{-1}(\omega) + l_{um}^{-1}(\omega) + l_{gb}^{-1}(\omega) + l_{res}^{-1}(\omega))^{-1} + l_{min} \quad \text{(S9)}$$

where l_{pd} is the inverse of the scattering time from point defects, l_{gb} is that of grain boundaries also incorporating extended structural motifs such as stacking faults, l_{res} is that of resonant effects (scattering of localized vibrations from the Einstein vibrations, as discussed in the heat capacity), l_{um} is the Umklapp scattering[S29], and l_{min} is the minimum mean free path that is allowed for the phonons and defines the lower limit of l_{ph}.

These are included by using each term's characteristic dependence on frequency (ω) shown in Eq. S10-S13.

$$l_{pd}^{-1}(\omega) = \frac{V\omega^4}{4\pi v_s^4}\left(\sum_i f_i\left(1-\frac{m_i}{\bar{m}}\right)^2 + \left(1-\frac{r_i}{\bar{r}}\right)^2\right) = A\cdot\omega^4 \quad \text{(S10)}$$

where V is the volume per atom, f_i is the fractional occupancy of atoms with mass m_i and radius r_i of a crystallographic position with average mass $\bar{m}$ and radius $\bar{r}$. The value of the point-defect scattering pre-factor A was calculated for $Ba_{10}Y_6Ti_4O_{27}$ (Table S5) and kept fixed during fitting of the thermal conductivity data.

$$l_{um}^{-1}(\omega) = B\cdot\omega^2\cdot T\cdot e^{\frac{-\theta_D}{3T}} \quad \text{(S11)}$$

$$l_{gb}^{-1}(\omega) = C\cdot\omega^2 \quad \text{(S12)}$$

$$l_{res}^{-1}(\omega) = \frac{D\cdot\omega_0^2\cdot\omega^2}{(\omega_0^2-\omega^2)^2+\Gamma^2\cdot\omega_0^2\cdot\omega^2} \quad \text{(S13)}$$

The parameters B, C, D and l_{min} were modified to model the data. The parameters v_s (speed of sound), θ_D (the Debye temperature) and ω_0 (the Einstein frequency) were obtained from experimental measurements of the Grüneisen parameter and heat capacity. Γ represents the damping factor of the localized mode, which is proportional to Λ/π, where Λ is the logarithmic decrement, and is refined as part of the model[S30]. The final parameters used to model the experimental thermal conductivity data of $Ba_{10}Y_6Ti_4O_{27}$ are given in Table S5. The contribution of the second Einstein term extracted from the heat capacity is very small and has a negligible effect on the fit, so is excluded from the model.

Using these parameters, the low temperature thermal conductivity (<100 K) is dominated by the C term, which is associated with extended structural motif scattering. Typically assumed to be a result of grain boundary scattering, this extended phonon scattering can also arise from defect aggregates[S31], stacking faults[S32], and dislocations[S33], or in this case the aperiodic nature of the structure[43,53,54] (in contrast to the other features listed, the motifs in aperiodic structure form part of the ordered atomic-scale structure of the structure itself, which is periodic in four dimensions and aperiodic but ordered in the three dimensional projection observed), and can result in a significant reduction in the phonon mean free path. In this model, the aperiodic structure is treated as a periodic three-dimensional structure that generates phonons and the modulations are extended structural motifs that scatter the phonons. As such, the aperiodic structure of $Ba_{10}Y_6Ti_4O_{27}$ provides a further route to reducing l_{ph} from phonon scattering processes associated with the structural modulation and aperiodic structure. At higher temperatures, the l_{min} term dominates, consistent with glass-like state thermal conductivity. However, all terms contribute, indicating that extended structural motif and resonant scattering mechanisms all play a role in phonon transport and therefore the thermal conductivity of the material. The additional resonant term (Eq. S13) is included to account for the low-frequency Einstein modes (θ_{E1}). The origin of the distinctive dip in the thermal conductivity at $T \approx 100$ K, and observed boson peak in the heat capacity of $Ba_{10}Y_6Ti_4O_{27}$ are both associated with resonance scattering processes[9]. These effects in combination with a relatively low speed of sound and Debye frequency, resulting from a multitude of bonding environments, enhanced further by structural modulation, lead to the exceptionally low value of the thermal conductivity. The behavior of the thermal conductivity as a function of temperature required two conduction terms for accurate modelling; one for the low frequency modes associated with θ_{D1} and one for the higher frequency modes described by θ_{D2}, with their respective Debye temperatures and different l_{min} values (l_{min1} and l_{min2}).

The value of 6.0(2) Å for l_{min1} is consistent with glass-like phonon conduction, associated with the higher frequency oxide modes (θ_{D2}), whereas the value of 2.5(2) Å for l_{min2} is very short and of the same order of magnitude as typical bond distances in $Ba_{10}Y_6Ti_4O_{27}$, and is associated with the lower frequency metal (Ba, Y) modes (θ_{D1}). This indicates that the lower frequency vibrations have a smaller than expected contribution to the thermal conductivity, which is modelled by a very small value of l_{min2}. This suggests that these modes may be heavily localized, further supporting the observation of a boson peak in the low temperature heat capacity, and may also participate in non-phononic conduction such as hopping[S34], or diffusion[S35]. This is consistent with the expected localization of vibrations in an aperiodic material.

Phonon calculations and comparison against Raman data

Γ point phonon calculations for the commensurate (t = 0) approximant model of $Ba_{10}Y_6Ti_4O_{27}$ were performed and are compared against the experimental Raman spectrum in Figure S8 The calculations show that no modes are expected above 800 cm^{-1}, which is supported by the absence of modes above this frequency in the measured Raman spectrum. Two main sets of phonons are observed in both theoretical and experimental spectra; lower frequency modes below 500 cm^{-1}, associated with heavier Ba, Y, and Ti cations, and higher frequency modes above 600 cm^{-1} which are typical of O vibrations.

There is good agreement between low-frequency contributions in the Γ point calculations and the Einstein frequencies (ω_{E1} = 66.7 cm^{-1} and ω_{E2} = 39.6 cm^{-1}) associated with the Einstein temperatures (θ_{E1} and θ_{E2}, Table S9) extracted from the experimental

heat capacity. This also agrees with the N_{E1}>>N_{E2} contributions that are obtained from fitting the experimental heat capacity, as the lower frequency contribution has a much smaller density of states (Figure S8, Table S9). The two Debye contributions ($\omega_{D1} \approx 202$ cm^{-1} and $\omega_{D2} \approx 593$ cm^{-1}) associated with the Debye temperatures (θ_{D1} and θ_{D2}, Table S9) extracted from the heat capacity represent Ba-O and Y/Ti-O bonding, respectively. This shows further agreement between measured and calculated parameters for $Ba_{10}Y_6Ti_4O_{27}$, and supports the need for multiple Einstein contributions for accurate modelling of the heat capacity. The group of high-frequency modes above 650 cm^{-1} have minimal contribution to the heat capacity and so are not represented by parameters extracted from the modelling.

It is noted that the experimental Raman spectra also highlight the absence of any unexpected modes in new $Ba_{10}Y_6Ti_4O_{27}$. Large alkaline earth cations such as Ba^{2+} will readily form highly stable compounds with large anions such as CO_3^{2-}. As seen in Figure S24, there is no evidence for the presence of carbonate or hydroxide containing species in the measured Raman spectrum of new $Ba_{10}Y_6Ti_4O_{27}$ indicating that the material is a pure oxide.

Supporting Figures

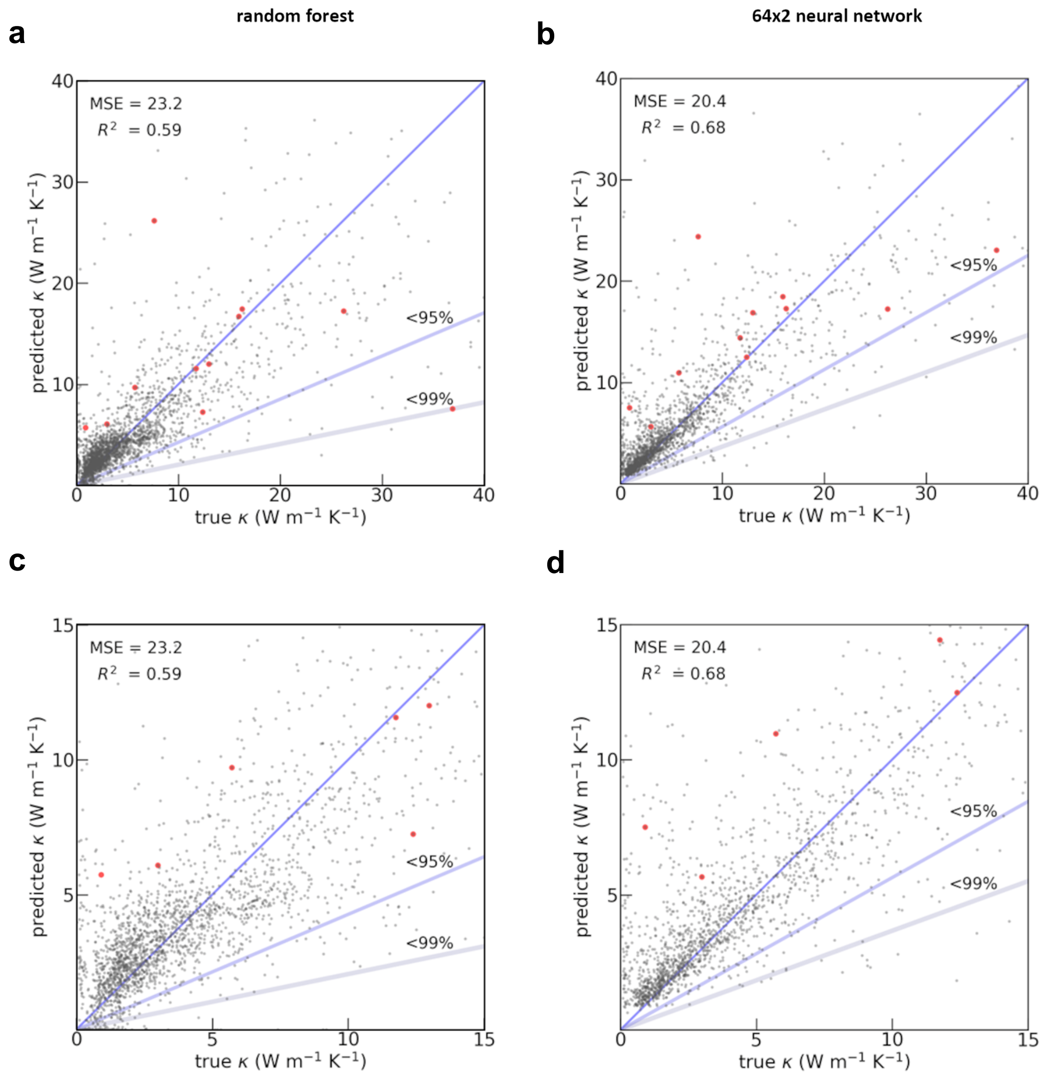

Figure S1 Comparison with example materials in the $Y^{3+} - Ba^{2+} - Ti^{4+} - O^{2-}$ phase diagram. (**a** and **b**) Full scale, and (**c** and **d**) expanded view of parity plots. Some known materials in the $Y^{3+} - Ba^{2+} - Ti^{4+} - O^{2-}$ phase field have calculated values of lattice thermal conductivity (highlighted in red), taken from the TEDesignLab dataset featured in the work of Gorai *et al.*[17] Quaternary materials in the face and ternaries along the edges of the phase field were not included in the training data, but were included in the testing set to help assess model performance. Specific compositions and their associated thermal conductivities are listed in Table S3. Cross validation performance is compared using the mean squared error (MSE) and the coefficient of determination (R^2) for random forest (**a** and **c**) and the neural network (**b** and **d**) models taking the average of 5 instances of k = 5 fold random cross validation. The darkest line shows the 1:1 correspondence of a perfect model, and lighter lines show the line that lies below 95% and 99% of all predictions, which gives an estimate of the chance of falsely predicting a low thermal conductivity (*i.e.,* a "false positive"). No models produce any predictions with $\kappa < 0$ W m^{-1} K^{-1}. The 64 × 2 neural network was used to construct the ternary plots shown in Figure 1c and 1d, as well as Figure S13.

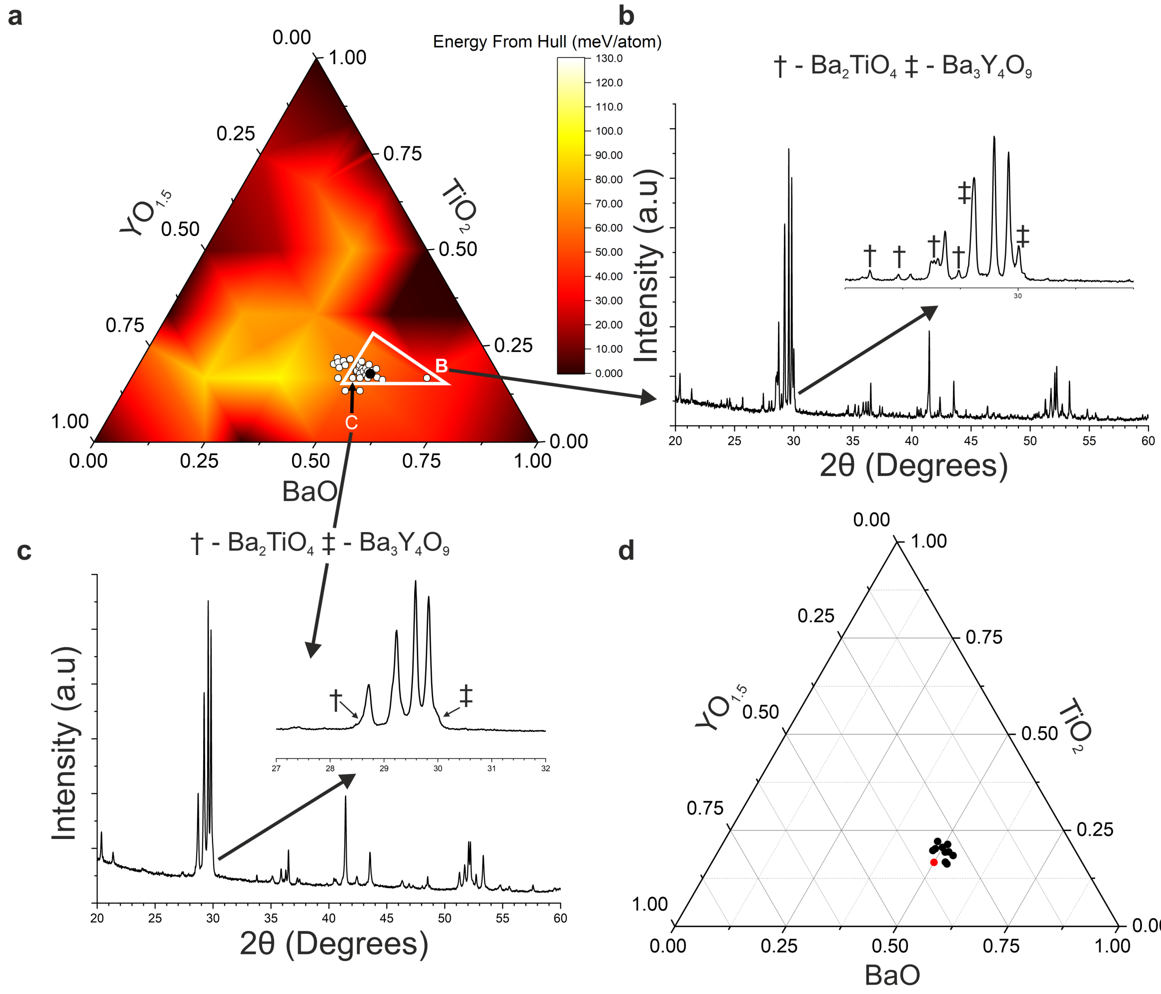

Figure S2. Initial synthetic screening and isolation of new $Ba_{10}Y_6Ti_4O_{27}$. **a**, Ternary plot of the 32 compositions sampled experimentally in the isolation of $Ba_{10}Y_6Ti_4O_{27}$ overlaid with the energy surface and white triangle plotted in Figure 1b which is the compositional target region identified by the combination of probe structure calculation and composition-based machine learning as most likely to contain new phases with low thermal conductivity. Labels 'B' and 'C' in (a) indicate the compositions that were synthesised first (the same compositions of $Ba_{0.667}Y_{0.167}Ti_{0.167}O_{1.25}$ and $Ba_{0.5}Y_{0.333}Ti_{0.167}O_{1.33}$ shown in Figure 1b and 1d). **b**, PXRD pattern of sample at the initial composition 'B', which is $Ba_{0.667}Y_{0.167}Ti_{0.167}O_{1.25}$, shows reflections from an unknown and known (Ba_2TiO_4 and $Ba_3Y_4O_9$) phases **c**, PXRD pattern of initial sample at composition 'C', which is $Ba_{0.5}Y_{0.333}Ti_{0.167}O_{1.33}$, with the majority of the observed reflections arising from the new unknown quaternary phase, and with fewer reflections from known phases compared to 'B'. **d**, EDX data collected on this initial sample 'C', revealing the presence of a new phase with the average composition $Ba_{0.50(4)}Y_{0.30(3)}Ti_{0.20(2)}O_{1.35(13)}$ from 10 particles, the nominal composition of $Ba_{0.5}Y_{0.333}Ti_{0.167}O_{1.33}$ is shown in red. A combination of these EDX and PXRD data guided the synthesis of a further 30 compositions shown by the points in (a) until a high purity sample was achieved at the nominal composition of $Ba_{0.53}Y_{0.29}Ti_{0.18}O_{1.32}$ indicated by the black point in (a) which is the composition used for structural characterisation and physical property measurements.

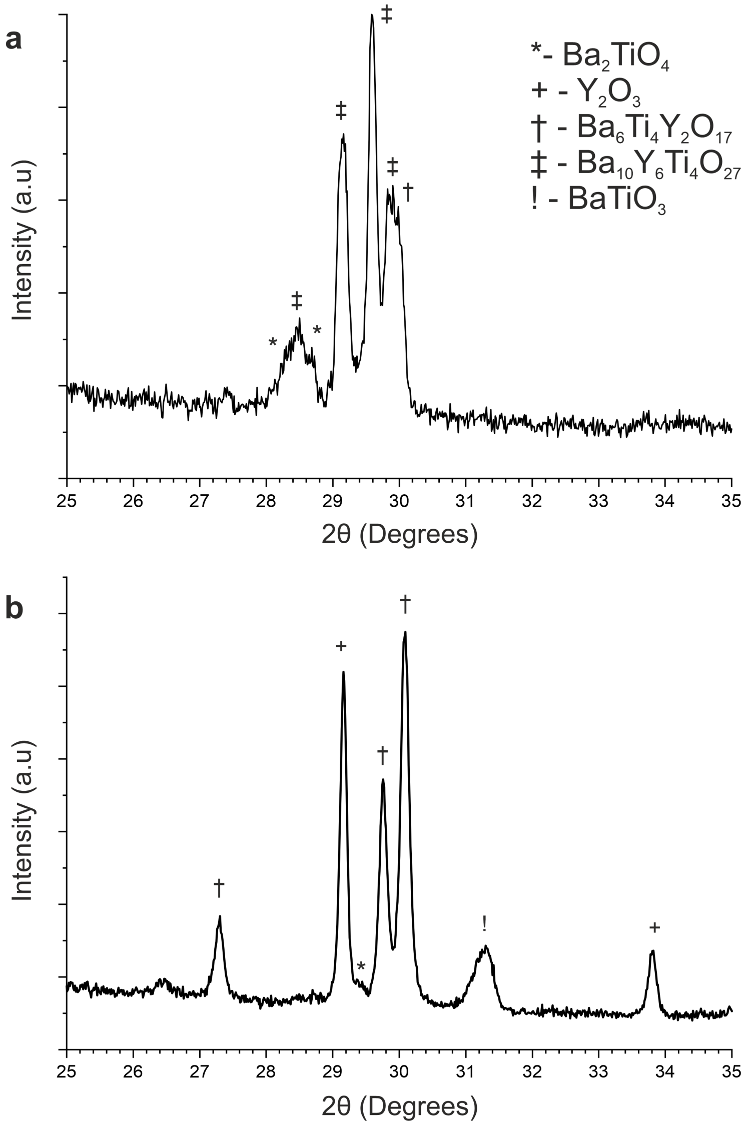

Figure S3. Decomposition of new $Ba_{10}Y_6Ti_4O_{27}$ under extended heating at the synthesis conditions or at higher temperature, suggesting metastability**.** **a**, PXRD pattern of a sample of the new phase $Ba_{10}Y_6Ti_4O_{27}$ that has been heated for an additional 8 hours at 1000°C. The PXRD pattern indicates that the sample has begun to decompose into Ba_2TiO_4 and $Ba_6Y_2Ti_4O_{17}$ shown by asterisks and single cross symbols respectively. **b**, PXRD pattern of a sample of $Ba_{10}Y_6Ti_4O_{27}$ that has decomposed into Ba_2TiO_4, Y_2O_3, $Ba_6Ti_4Y_2O_{17}$ and $BaTiO_3$ after an initial attempt to process the material to density by SPS at high temperature (heating to 1300°C at a pressure of 60 MPa for 3 minutes). PXRD patterns were collected using Cu Kα_1 radiation (λ = 1.54056 Å).

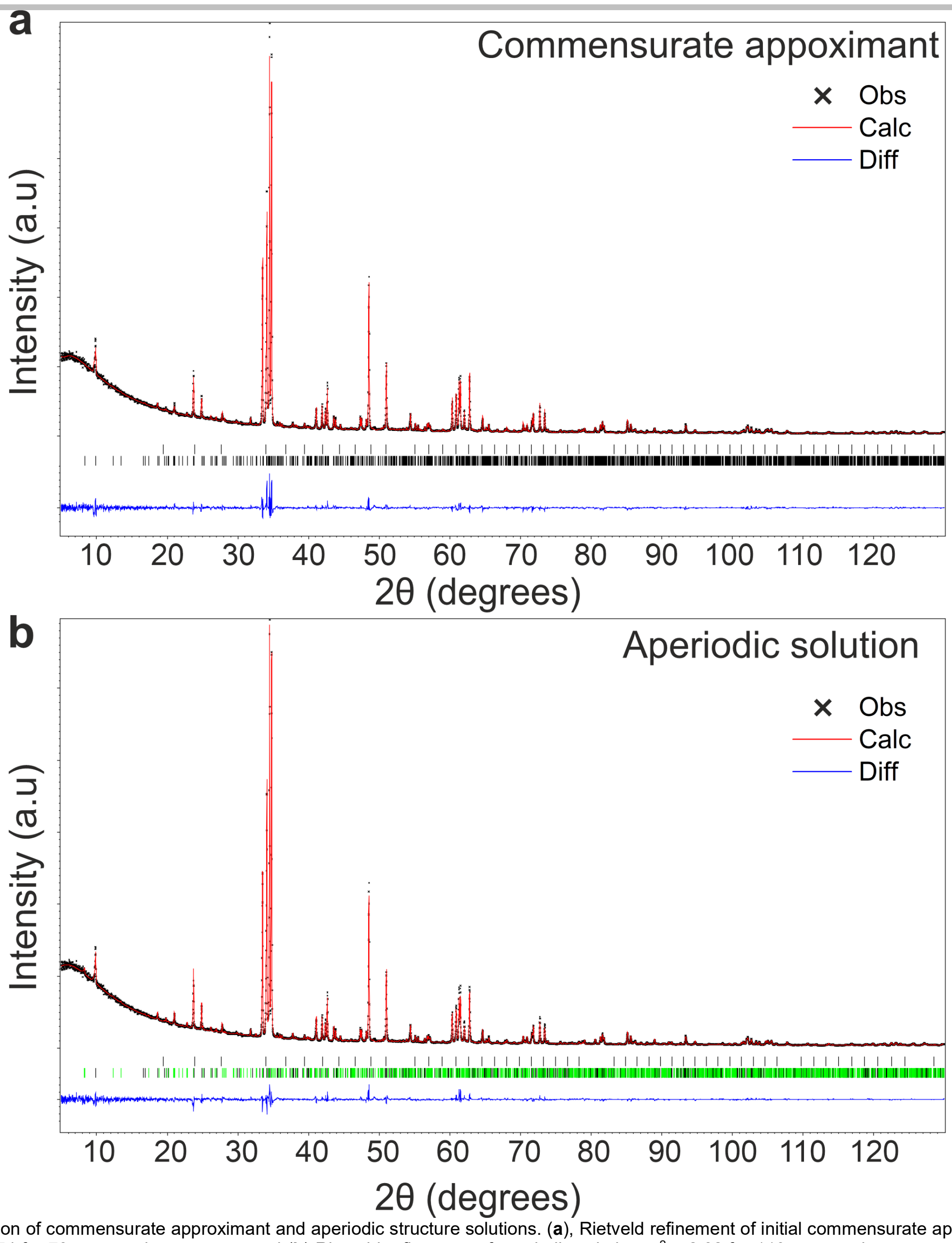

Figure S4. Comparison of commensurate approximant and aperiodic structure solutions. (**a**), Rietveld refinement of initial commensurate approximant solution for $Ba_{10}Y_6Ti_4O_{27}$, χ^2 = 3.54 for 73 structural parameters and (**b**) Rietveld refinement of aperiodic solution, χ^2 = 2.62 for 110 structural parameters. Both fits presented here are against the same laboratory PXRD data with λ = 1.78901 Å (Co Kα_1). In both plots, the top set of tick marks indicate reflections for Y_2O_3: top, refined to 1.46(4) mol%, bottom 2.78(7) mol% - the difference is as a result of changing between models for $Ba_{10}Y_6Ti_4O_{27}$ as most of the reflections for Y_2O_3 overlap with $Ba_{10}Y_6Ti_4O_{27}$, in the final model presented in the main text, the Y_2O_3 content was refined to 1.7(10) mol%. The bottom set of tick marks indicate the reflections for $Ba_{10}Y_6Ti_4O_{27}$, green tick marks in (b) indicated the position of satellite reflections from the structural modulation.

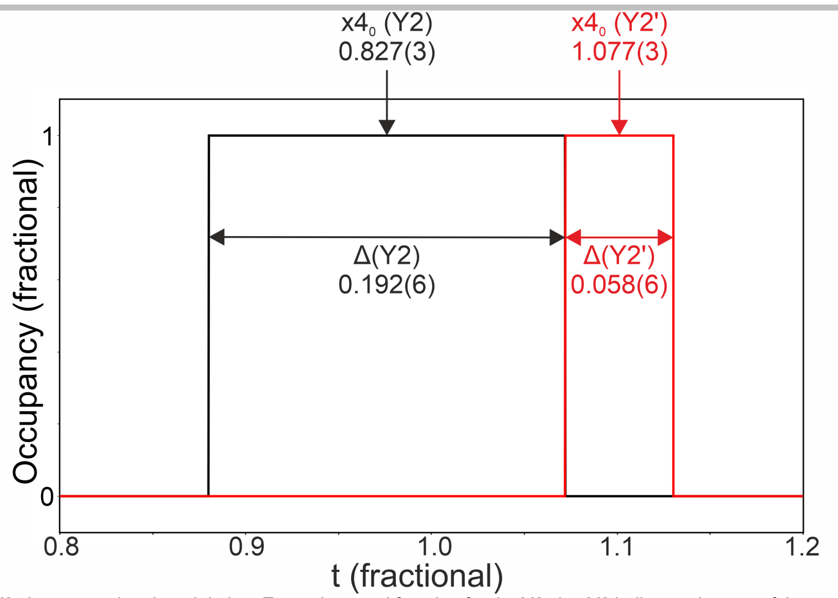

Figure S5 Crenel function for Y2 site occupational modulation. Example crenel function for the Y2 site, Y2 indicates the part of the crenel occupied by yttrium (black) and Y2′ by titanium (red). Each part of the site has a $x4_0$ and Δ parameter, where $x4_0$ indicates the center point of the crenel along t and Δ is the width of the occupied component of the crenel, with their refined values displayed. In the refined model, the total Δ is constrained to be equal to ¼ (one fully occupied site) and the $x4_0$ parameters constrained so that the two components of the site follow on from each other along t. The total crenel illustrated here then corresponds to one of the mixed yttrium – titanium crenels shown in Figure 4a and Figure S6a and b.

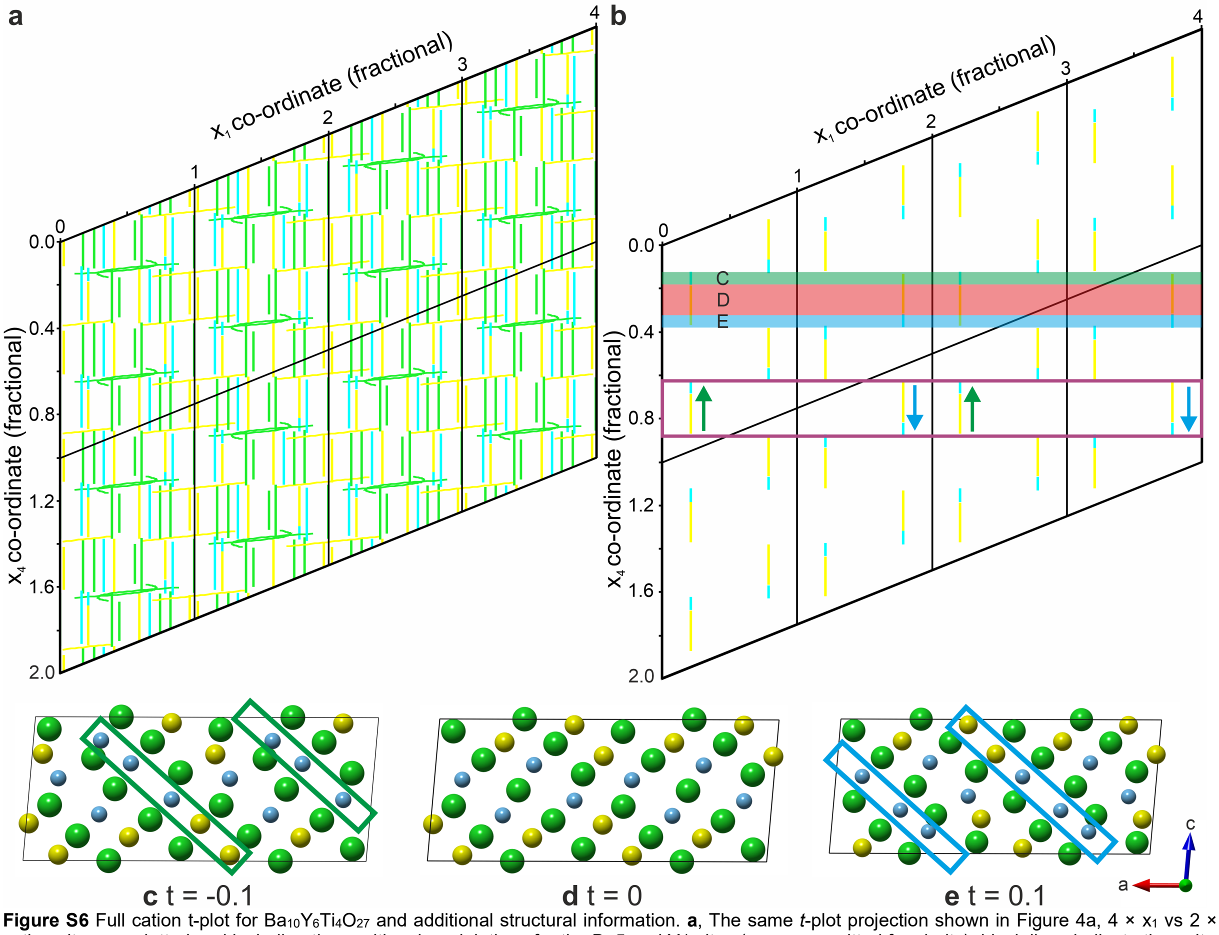

Figure S6 Full cation t-plot for $Ba_{10}Y_6Ti_4O_{27}$ and additional structural information. **a**, The same *t*-plot projection shown in Figure 4a, 4 × x_1 vs 2 × x_4: here all 11 cation sites are plotted and including the positional modulations for the Ba5 and Y1 sites (oxygens omitted for clarity), black lines indicate the unit cell boundaries. **b**, t-plot of the x_1 vs. x_4 plane for the Y2 site which switches occupancy between Y and Ti. The three colored blocks indicate the types of cation ordering observed in the structure as a result (**c**) (green block) cation motif at t = -0.1, where the first half of the Y2 sites are replaced with Ti, with the rows highlighted with the green box, (**d**) (red block) the cation motif at t = 0 and (**e**) (cyan block) the cation motif at t = 0.1 where the second half of the Y2 sites are replaced by Ti. As only half of the Y2 sites are replaced with Ti for any given slice of the structure, while 20% of the Y2 sites are Ti, this corresponds to 40% of the structure that has either of the two substitutions shown in (c) and (e). The purple box is the next block of the structure with the same origin as the shaded region, where the arrows highlight the part of the Y2 crenel containing Ti, the green arrows result in motif (c) and the cyan arrows result in motif (e). Projections which do not pass through either set of Ti crenels will result in the motif with no substitution (d) Cations colored as follows: Ba (green), Y(yellow) and Ti (cyan).

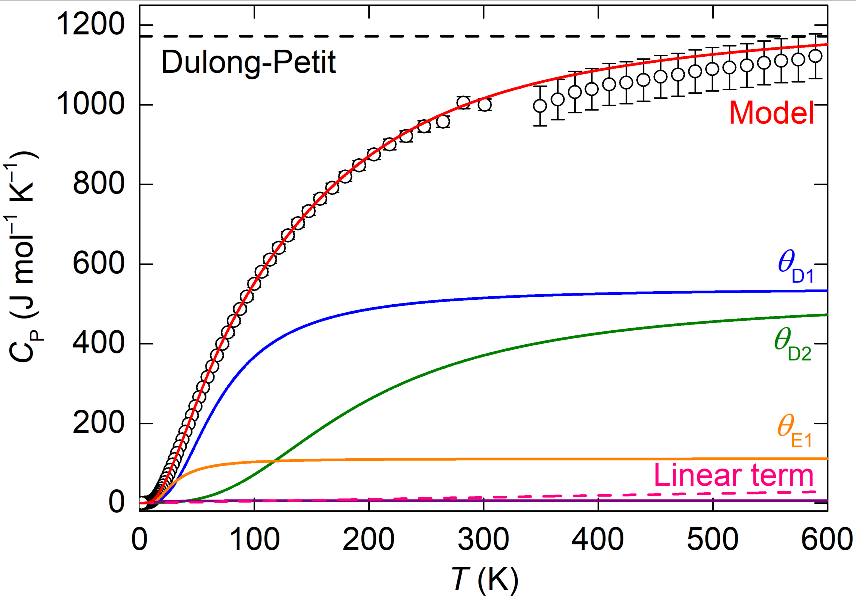

Figure S7 Modelling the heat capacity of $Ba_{10}Y_6Ti_4O_{27}$. Fit to the heat capacity of $Ba_{10}Y_6Ti_4O_{27}$, showing the contribution from the two Debye terms (θ_{D1} and θ_{D2}), two Einstein terms (θ_{E1} and θ_{E2}), and the linear term shown as the dashed pink line. The contribution of θ_{E2} is very small and is shown by the solid purple line. The linear term is 25(1) mJ mol^{-1} K^{-2}.

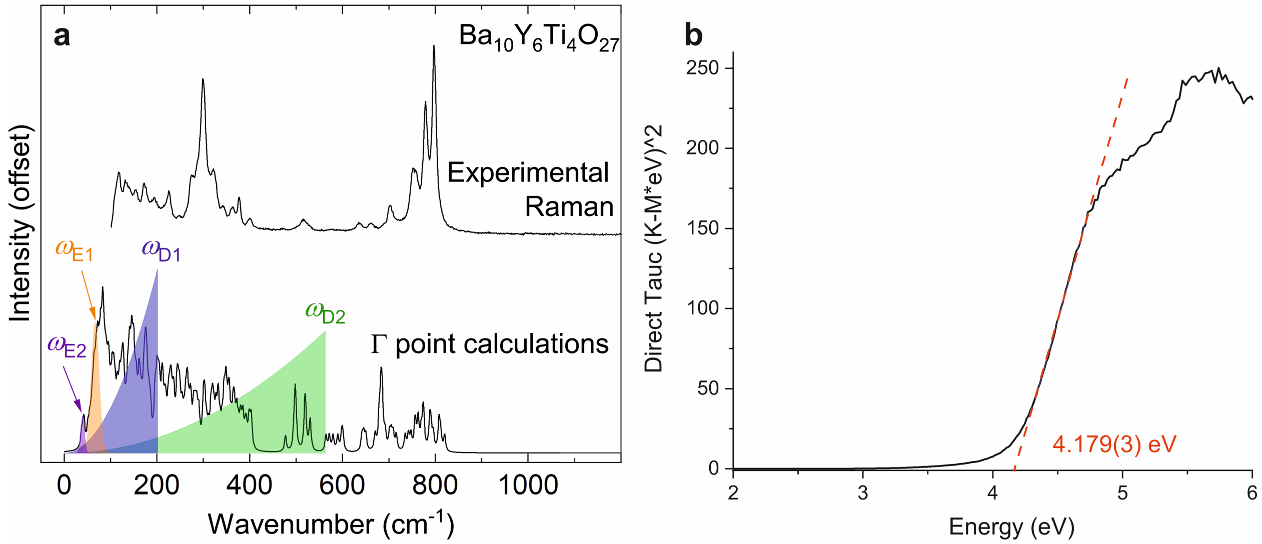

Figure S8 a. Raman spectrum of $Ba_{10}Y_6Ti_4O_{27}$. Measured Raman spectrum of $Ba_{10}Y_6Ti_4O_{27}$ (upper line) and the computed phonon DOS (described on p3 and discussed on p9 of this document) produced from Γ point phonon calculations (lower line) compared against the DOS contributions from the Debye and Einstein frequencies (ω_D and ω_E) derived from the Debye and Einstein temperatures (θ_D and θ_E) extracted from fitting the experimental heat capacity (Figure S7, Table S9). Blue and green shading corresponds to the Debye DOS contributions up to ω_{D1} and ω_{D2}, respectively, while orange and purple peaks at low frequencies represent the two Einstein contributions ω_{E1} and ω_{E2}. **b**. Tauc plot of $Ba_{10}Y_6Ti_4O_{27}$ band gap calculated using the Kubelka-Munk function obtained from diffuse reflectance measurement confirming the electronically insulating nature of the material, with the measured bandgap of 4.179(3) eV.

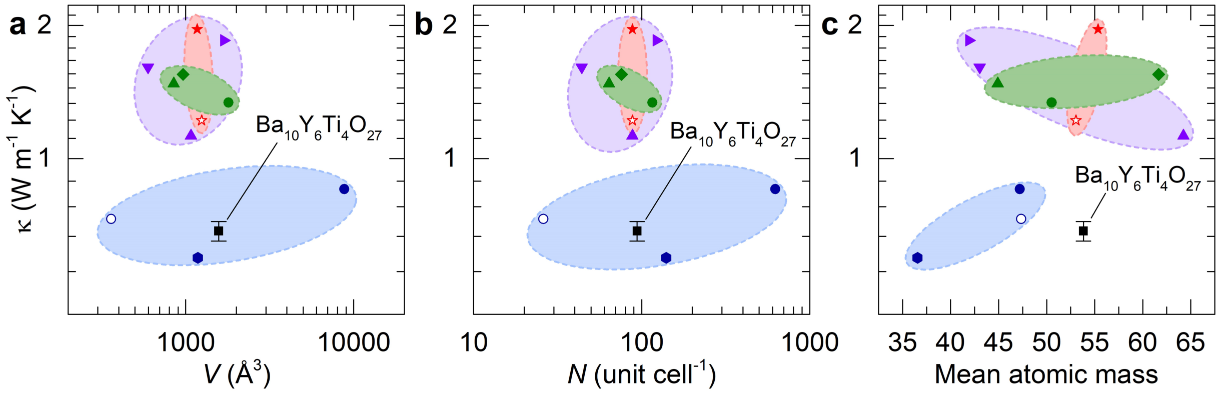

Figure S9 Thermal conductivities of low κ (< 2 W m^{-1} K^{-1}) oxide materials at 373 K against structural parameters. Comparison of thermal conductivities of oxide materials with κ_{latt} below 2 W m^{-1} K^{-1} against $Ba_{10}Y_6Ti_4O_{27}$ (black square) plotted as a function of **a**, unit cell volume, **b**, number of atoms within the unit cell, and **c**,

mean atomic mass[31-39]. Shaded areas correspond to the framework cation: niobates (purple), zirconates (red), molybdates (blue) and titanates (green). Symbols match those used in Figure 5a of the main text. The functional outperformance of $Ba_{10}Y_6Ti_4O_{27}$ over other materials is clear through this comparison based on simple structural parameters; $Ba_{10}Y_6Ti_4O_{27}$ as a titanate has a thermal conductivity comparable to the best molybdates.

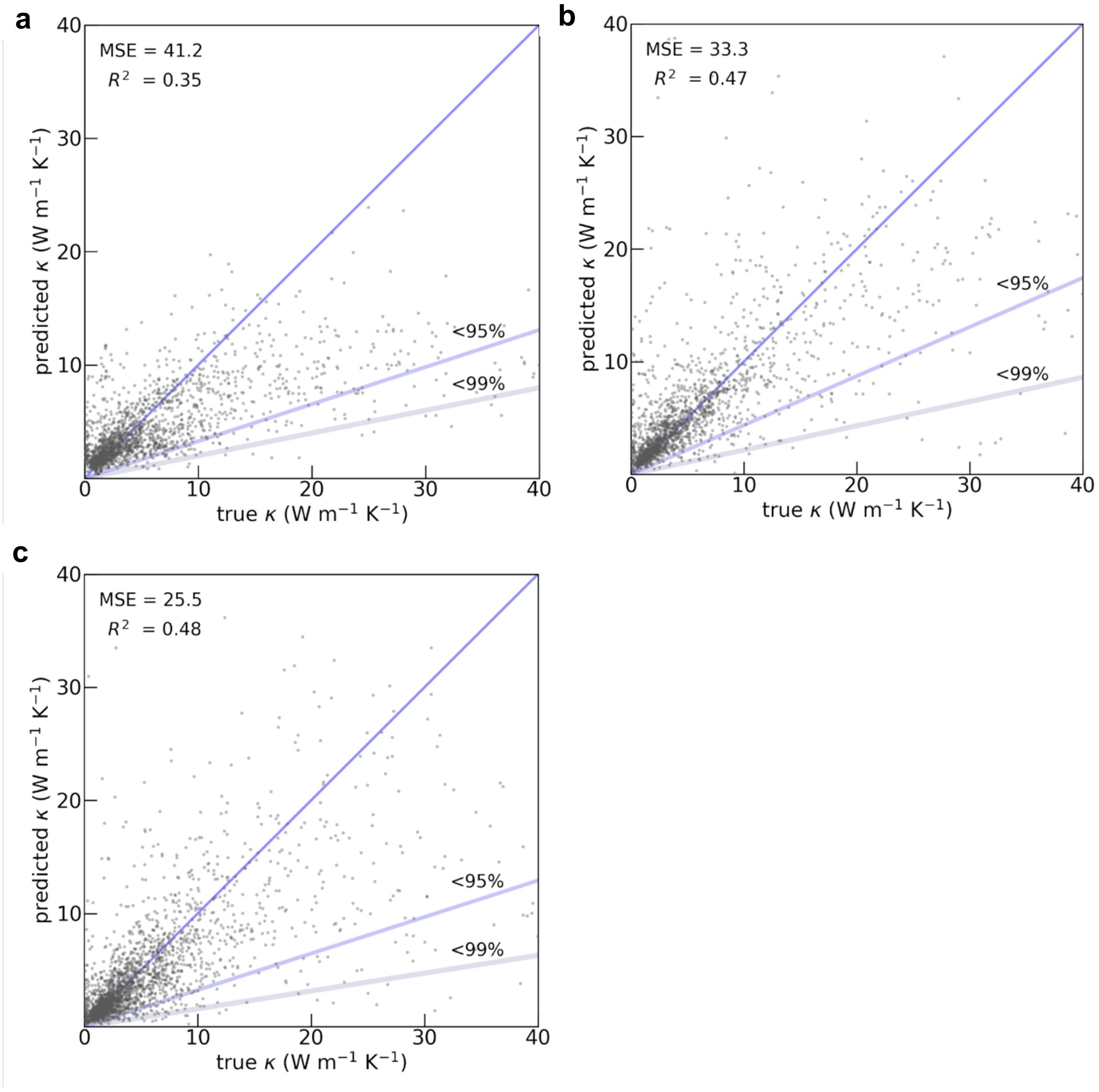

Figure S10 Thermal conductivity prediction using naive models and no engineered features. Simple models to predict thermal conductivity were constructed as benchmarks to assess the comparative performance of more complex models, using the MSE and R^2. The darkest line shows the 1:1 correspondence of a perfect model, and lighter lines show the line that lies below 95% and 99% of all predictions, which gives an estimate of the chance of falsely predicting a low thermal conductivity (*i.e.,* a "false positive"). Here, one-hot encoding of the elemental composition is used as the input, with no complex features, and performance is evaluated using the mean of 5 instances of k = 5 cross validation. (**a**), A linear regression on the logarithm of κ creates an extremely simple log-linear model, though it suffers from a large number of "false positives" (where the predicted thermal conductivity is low, but the true thermal conductivity is high). (**b** and **c**) A random forest and (c) a 64 × 2 ReLU neural network is increasingly complex, and improves the performance. For one-hot encoding of the elemental composition, the 64 × 2 neural network shows the best performance in terms of both MSE and R^2. Using engineered features increases the performance further, as in Figure S12.

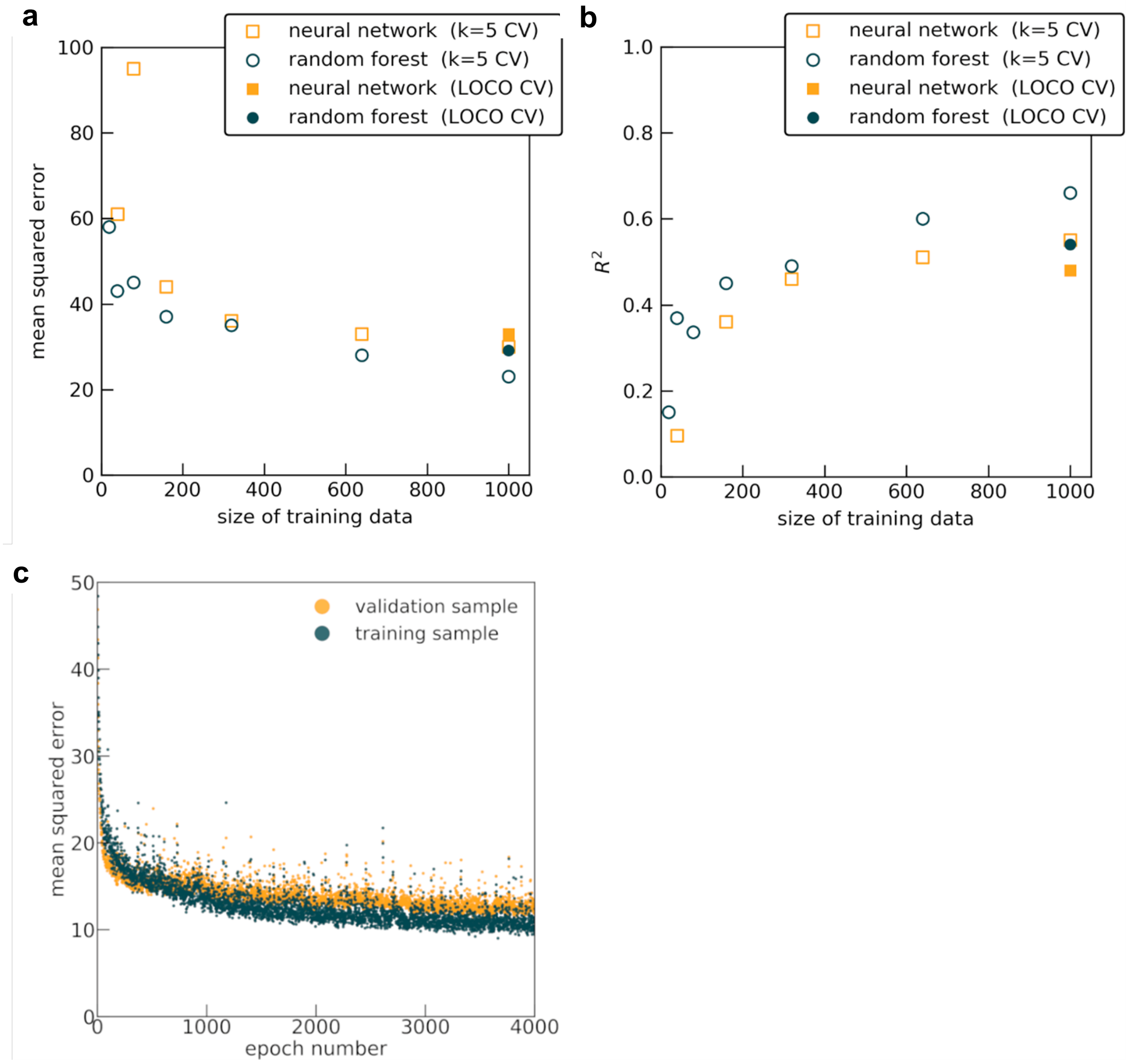

Figure S11 Effect of dataset size on model performance, and mean square error per epoch. Model performance improves with increasing size of dataset, as measured by the (**a**) mean squared error (MSE), and (**b**) the coefficient of determination (R^2). Random forests consistently outperform neural networks at comparable dataset sizes, particularly when using leave-one-cluster-out cross validation (LOCO CV) compared to k = 5 cross validation (k = 5 CV). The unsupervised clustering means that a consistent data subset cannot be used in the LOCO CV as the dataset size is varied, so the LOCO CV performance is evaluated by only considering the 1000-point training sample. The loss in performance is more clearly seen when using LOCO CV, and is particularly large for neural networks. (**c**) Training of a 64 × 2 neural network is smooth with no significant over-training. Further, the MSE is similar between training and validation datasets, suggesting the model generalizes well. With a patience of 500 epochs without a lowest overall validation loss, training comes to a halt. This monotonic training is consistent with a model that is primarily log linear in nature.

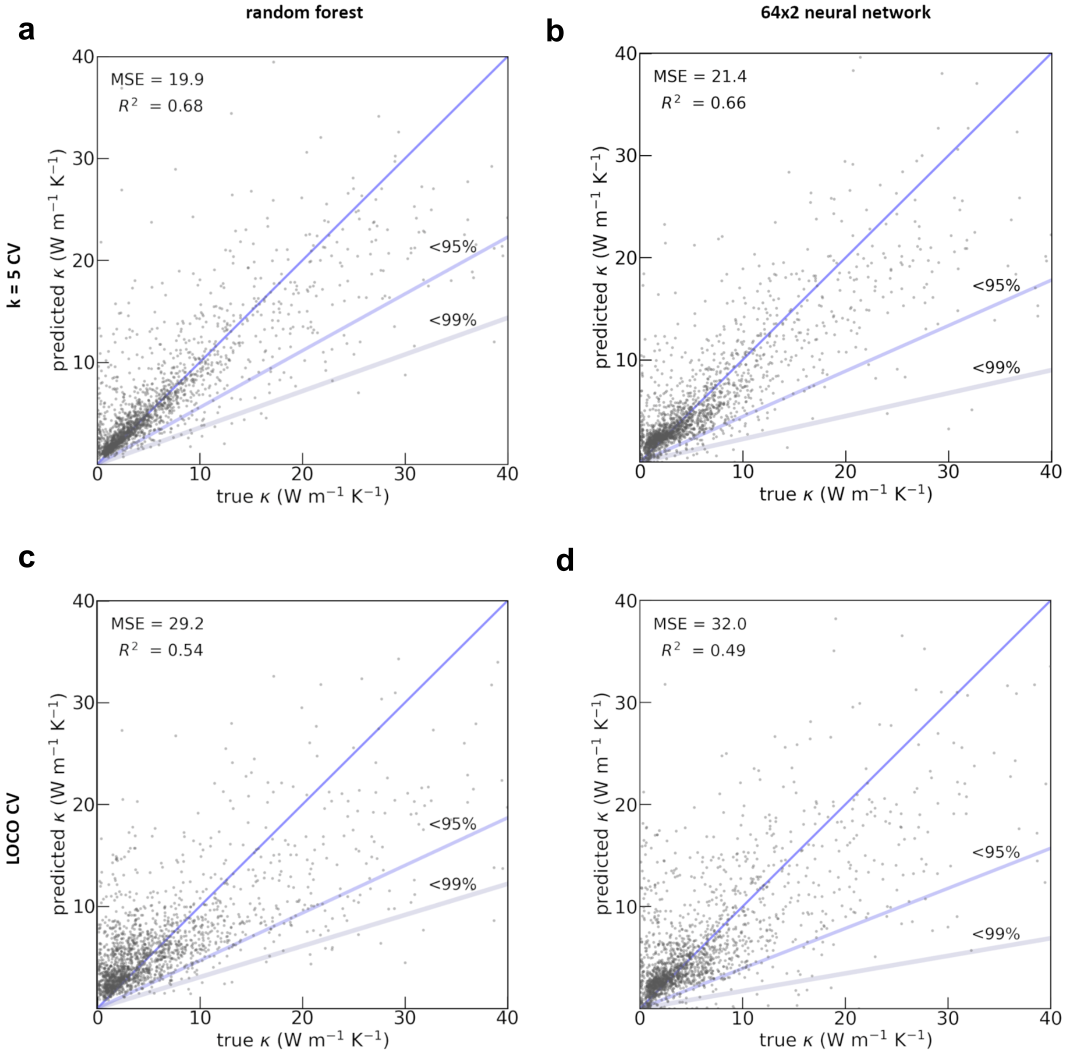

Figure S12 The influence of algorithm choice and cross validation regime on model performance. Cross validation performance is compared using the mean squared error (MSE) and the coefficient of determination (R^2) for random forest (**a** and **c**) and the neural network (**b** and **d**) models using both k = 5 fold random cross validation (**a** and **b**) and leave one cluster out cross validation, taking the average of 5 cycles of training and prediction, LOCO CV (**c** and **d**). The darkest line shows the 1:1 correspondence of a perfect model, and lighter lines show the line that lies below 95% and 99% of all predictions, which gives an estimate of the chance of falsely predicting a low thermal conductivity (*i.e.,* a "false positive"). The random forest outperforms the neural network for both forms of cross validation. The random cross validation represents the interpolation performance of the model, whereas the cluster cross validation gives a better measure of extrapolation performance. When extrapolating to unseen clusters, the models are more likely to predict "false negatives," where materials with low *κ* are predicted to have a high thermal conductivity. This not as problematic for screening in this application, where significant resources will be invested for a potentially interesting material, so avoiding false positives (materials with high *κ* but predicted to be low) is a high priority. No models produce any predictions with $\kappa < 0$ W m^{-1} K^{-1}. The 64 × 2 neural network was used to construct the ternary plots shown in Figure 1c and 1d, as well as Figure S13.

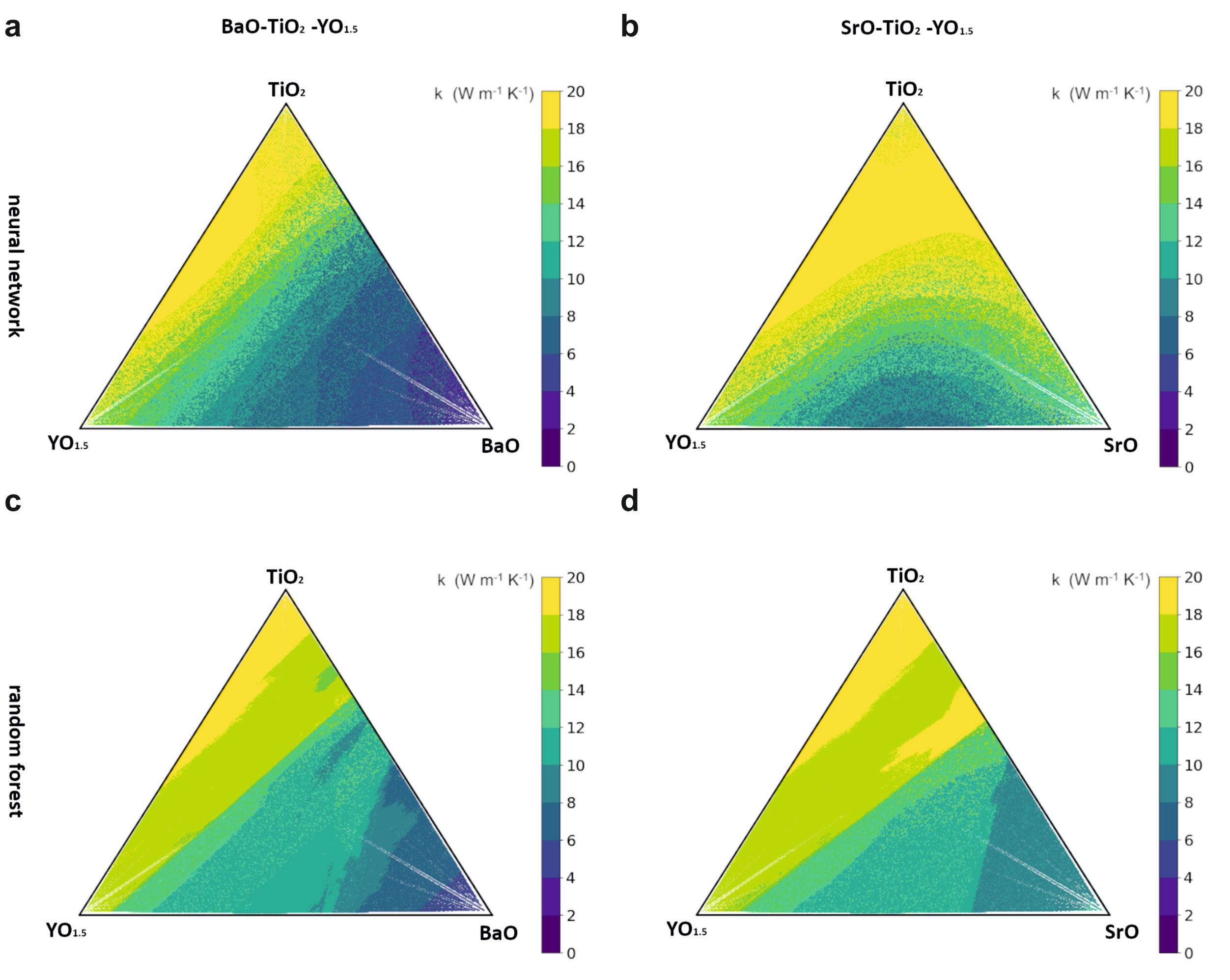

Figure S13 Estimating the thermal conductivity of phase diagrams based on the current knowledge frontier. 64 × 2 neural network model prediction of the thermal conductivity of the (**a**) $Y^{3+} - Ba^{2+} - Ti^{4+} - O^{2-}$ phase diagram and (**b**) $Y^{3+} - Sr^{2+} - Ti^{4+} - O^{2-}$ phase diagram. These are the same data used to construct Figure 1c and 1d, but shown here in a perceptually uniform color scheme to allow better quantitative evaluation and comparison with the random forest models. No data points in the face or on the edges of the phase field were used to train the models. The values of the predictions are quantitatively similar to the random forest model predictions (**c** and **d**), though the random forests produce noticeable discrete regions rather than a continuously differentiable function produced by the neural network. The average of 5 models is used for these plots to stabilise the output.

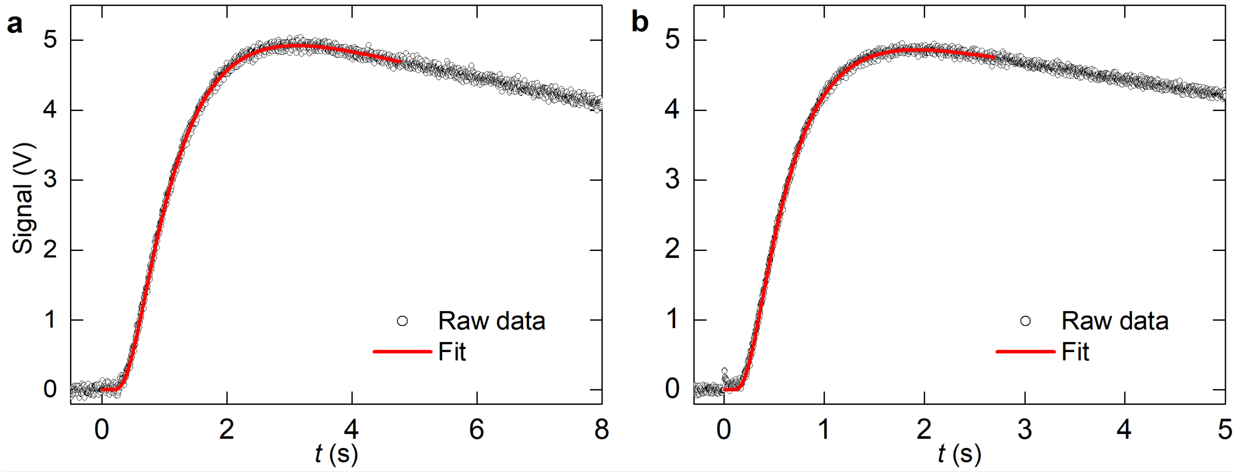

Figure S14 Fitting of raw data obtained from laser flash method. a-b, Red lines are the fits using the Cowan model[S23] to the raw data (black circles) at 297 K for (a) $Ba_{10}Y_6Ti_4O_{27}$ and (b) $Ba_6Y_2Ti_4O_{17}$.

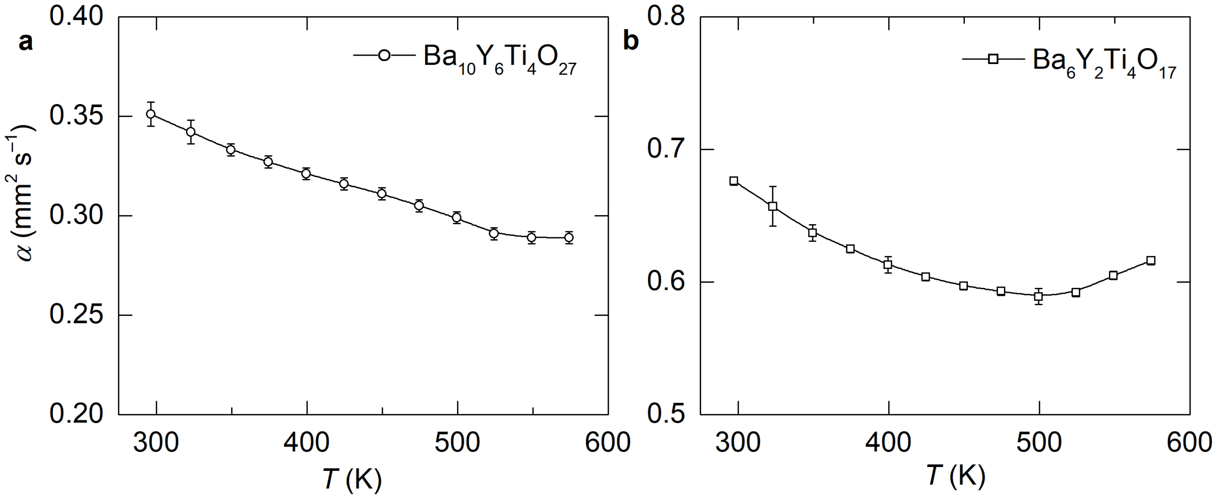

Figure S15 Thermal diffusivities obtained from laser flash analysis. a-b, Thermal diffusivities (*α*) of (a) $Ba_{10}Y_6Ti_4O_{27}$ and (b) $Ba_6Y_2Ti_4O_{17}$ measured in the temperature range 297 to 600 K. Error bars represent 3 e.s.d. of the measured points

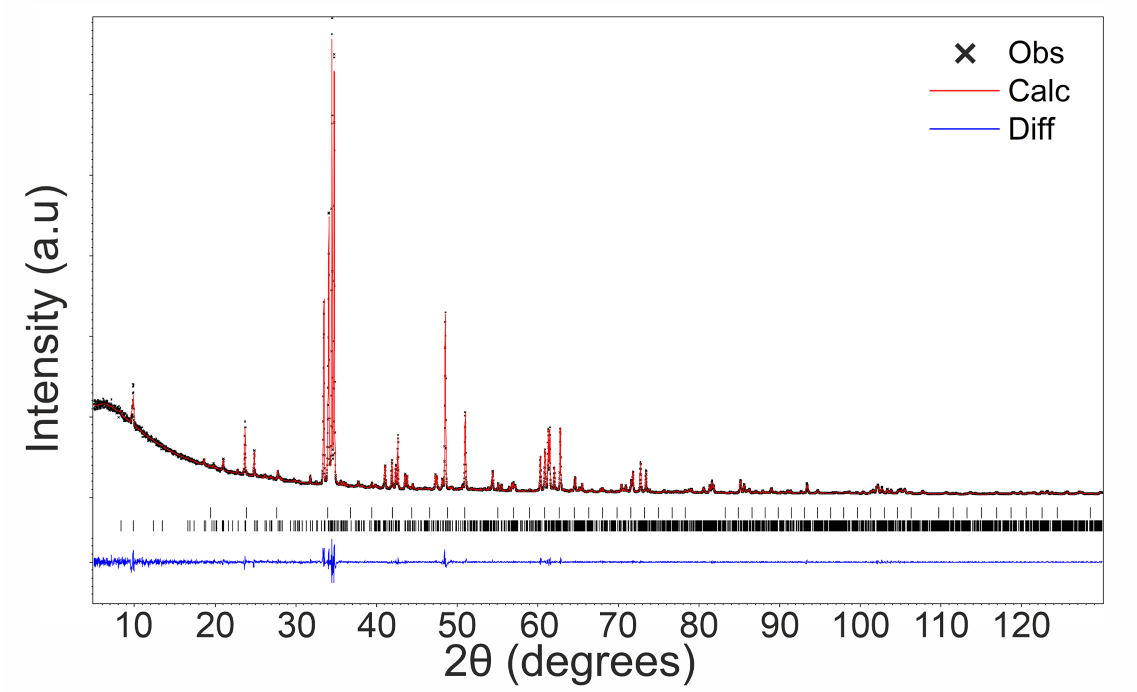

Figure S16 Initial Le Bail fit to lab-PXRD data. Le Bail fit to laboratory PXRD data for the commensurate approximant unit cell with a χ^2 of 1.82 with 47 parameters, used as the starting point for the structure solution of $Ba_{10}Y_6Ti_4O_{27}$. The upper tick marks are reflections for Y_2O_3 and the lower are for $Ba_{10}Y_6Ti_4O_{27}$.

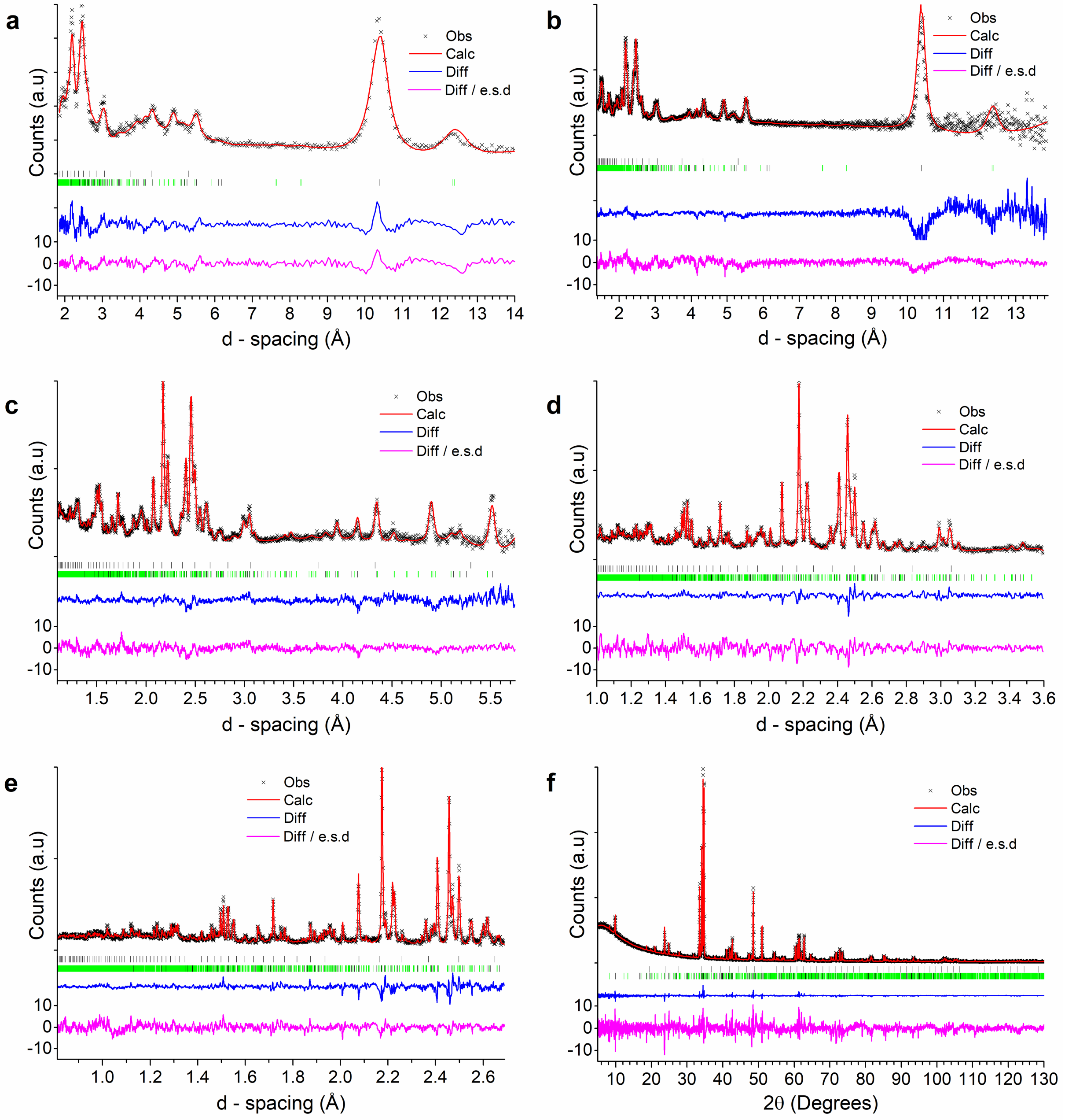

Figure S17 Initial modulated structure solution of $Ba_{10}Y_6Ti_4O_{27}$. Rietveld refinement of the initial modulated solution for $Ba_{10}Y_6Ti_4O_{27}$ with a combined $\chi^2 = 2.18$ for 110 structural parameters. a-e, data from detector banks 1 – 5 from the POLARIS beamline at the ISIS neutron source. f, Laboratory PXRD used in the combined refinement. The top row of tick marks indicate reflections for Y_2O_3, the bottom row for $Ba_{10}Y_6Ti_4O_{27}$, black tick marks are for sub-cell reflections, green for satellite reflections. The blue curve is the difference between the observed and calculated data. The magenta curve for each data set is the difference between the observed and calculated data divided by the e.s.d. on each observed data point.

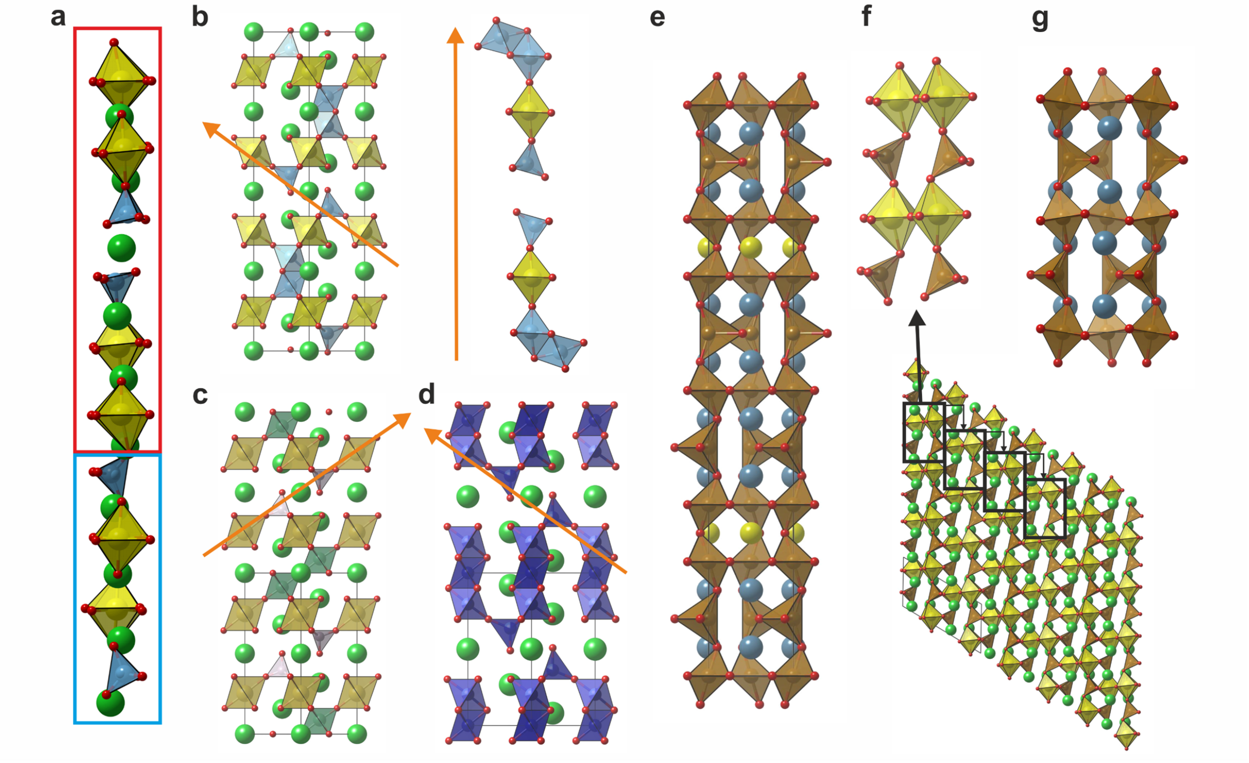

Figure S18 Structural motifs $Ba_{10}Y_6Ti_4O_{27}$ related to known materials. **a**, The 10a_p polyhedron ordering along the stacking axis of the pseudo cubic model of the structure of $Ba_{10}Y_6Ti_4O_{27}$ (as shown in Figure 2d and e), the red section of the stacking sequence is related to motifs observed in hexagonal perovskites and the cyan section related to Brownmillerite structures (which are themselves related to cubic perovskites). **b-d**, Examples of hexagonal perovskites with stackings related to the cyan region in (a): $Ba_6Y_2Ti_4O_{17}$[12], $Ba_6Na_2Nb_2P_2O_{17}$[S36] and $Ba_5TiCo_4O_{14}$[S37], when the polyhedron stacking is extracted along the same direction as (a) (highlighted with the orange arrows and the sequence extracted for (b)), they all contain the same terminal tetrahedral feature as $Ba_{10}Y_6Ti_4O_{27}$. **e-g**, examples of Brownmillerite related structures related to the red section highlighted in (a): $YCa_4Fe_5O_{13}$[S38], Ba_2FeYO_5[S39] and $Ca_2Fe_2O_5$[S40], containing polyhedral stacks alternating octahedra and tetrahedra. Unlike $Ba_{10}Y_6Ti_4O_{27}$, typically an odd number of octahedra along the stacking direction is required before the tetrahedral chains switch orientation in Brownmillerites. **f**, Ba_2FeYO_5 is another example of a perovskite-related superstructure containing shifts between stacks of polyhedra, where each adjacent stack is shifted by one perovskite layer, compared to four layers in $Ba_{10}Y_6Ti_4O_{27}$ highlighted in Figure 2e.

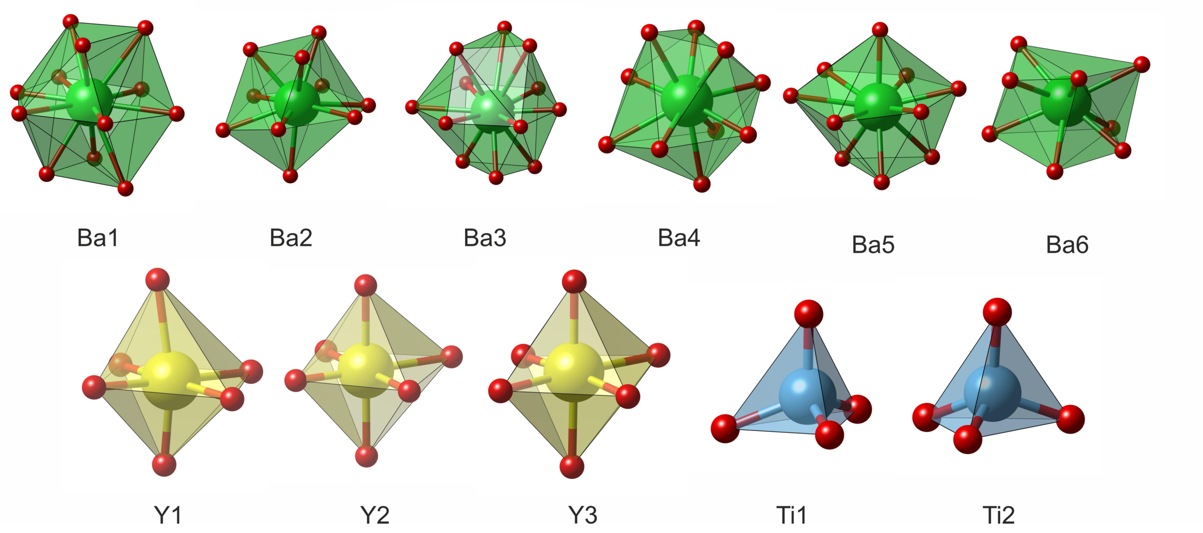

Figure S19 Cation co-ordination environments in $Ba_{10}Y_6Ti_4O_{27}$. Co-ordination polyhedra in $Ba_{10}Y_6Ti_4O_{27}$. The M–O co-ordination environments for the 4 × 1 × 1 supercell at t = 0 shown in Figure 2d, where M = Ba, Y, Ti. Atoms colored as follows: Ba (green), Y (yellow), Ti (cyan) and O (red)

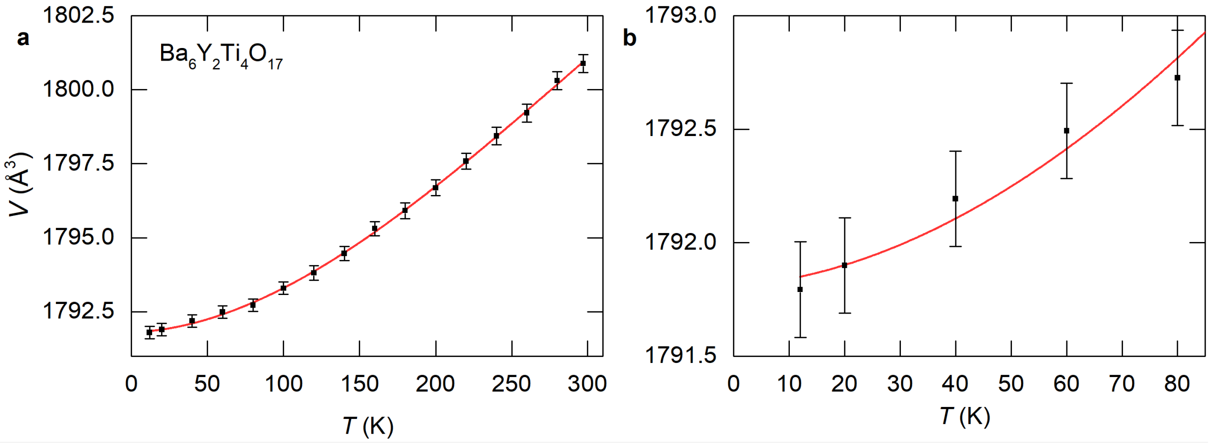

Figure S20 $Ba_6Y_2Ti_4O_{17}$ unit cell volume as a function of temperature. **a**, Variation in unit cell volume (*V*) as a function of temperature for $Ba_6Y_2Ti_4O_{17}$ obtained from Rietveld refinement. **b**, enlargement of the low temperature region. The red line represents the fitted polynomial (R^2 = 0.9994) detailed in Table S4 used to extract the volumetric thermal expansion coefficient (α_V).

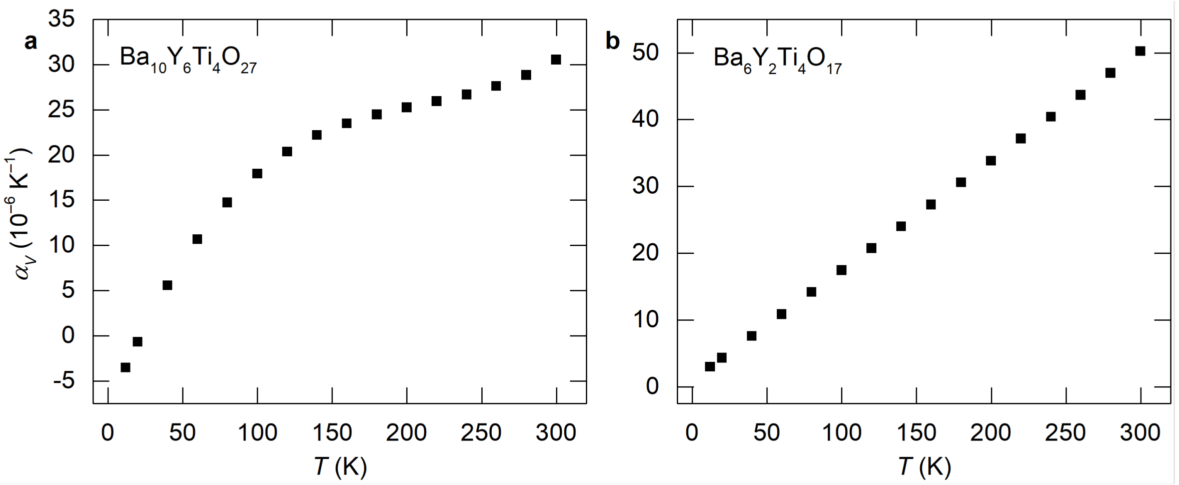

Figure S21 Volumetric thermal expansion coefficients. a-b, Thermal expansion coefficients (α_V) extracted from unit cell volumes as a function of temperature for (a) $Ba_{10}Y_6Ti_4O_{27}$ and (b) $Ba_6Y_2Ti_4O_{17}$.

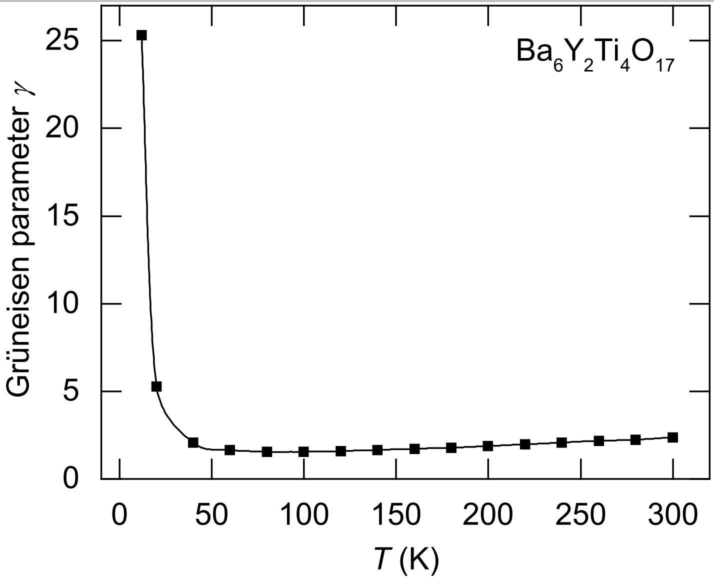

Figure S22 Grüneisen parameter of $Ba_6Y_2Ti_4O_{17}$. Temperature dependence of the Grüneisen parameter (*γ*) for $Ba_6Y_2Ti_4O_{17}$ obtained from volumetric thermal expansion coefficient (α_V), bulk modulus (B_T), molar volume (V_m) and heat capacity (C_V).

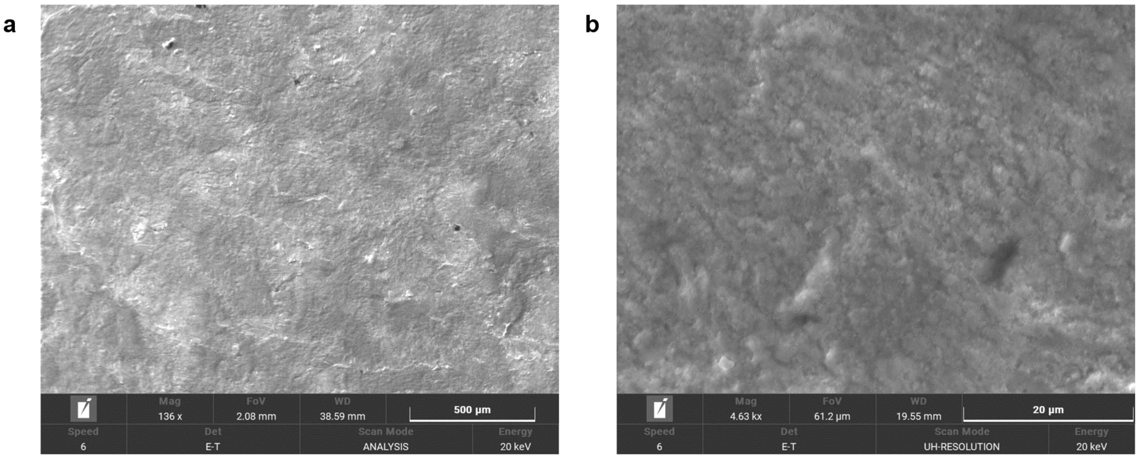

Figure S23 Porosity of ceramic bodies. **a-b**, Scanning electron micrograph (SEM) images taken at 136× and 4630× magnification from unpolished surfaces of $Ba_{10}Y_6Ti_4O_{27}$ dense ceramics highlighting minimal porosity.

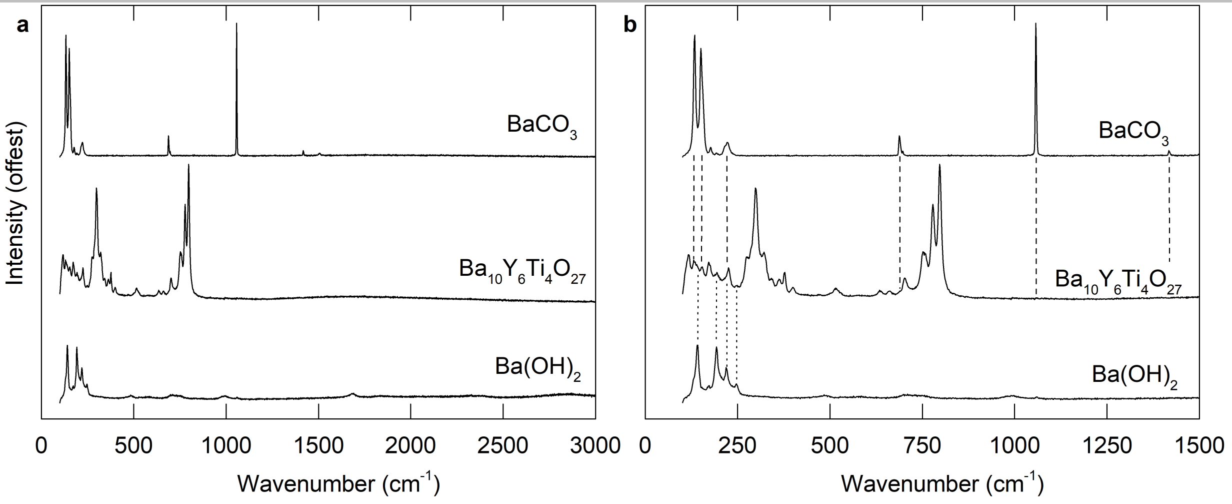

Figure S24 Raman spectra of $Ba_{10}Y_6Ti_4O_{27}$. **a**, Comparison of measured Raman spectra of the new quaternary $Ba_{10}Y_6Ti_4O_{27}$, $BaCO_3$ and $Ba(OH)_2$ clearly highlighting the absence of carbonate- and hydroxide-containing species in new $Ba_{10}Y_6Ti_4O_{27}$. Vertical dashed and dotted lines in (**b**) act as a guide to the eye only.

Supporting Tables

Table S1 Compositions used in the Y^{3+} - Ba^{2+} - Ti^{4+} - O^{2-} phase field stability calculations.

Composition	Normalized composition	Energy from convex hull (meV atom^{-1}) from DFT- this work	Source of structural model
**$Y_2Ti_6O_{15}$**	**$Y_{0.25}Ti_{0.75}O_{1.875}$**	22	Probe Structure
**$Y_6Ti_2O_{13}$**	**$Y_{0.75}Ti_{0.25}O_{1.625}$**	60	Probe Structure
**$Ba_2Y_2Ti_8O_{21}$**	**$Ba_{0.167}Y_{0.167}Ti_{0.667}O_{1.75}$**	50	Probe Structure
$BaY_2Ti_3O_{10}$	$Ba_{0.167}Y_{0.333}Ti_{0.5}O_{1.667}$	19	Probe Structure[14]
$Ba_2Y_6Ti_4O_{19}$	$Ba_{0.167}Y_{0.5}Ti_{0.333}O_{1.583}$	74	Probe Structure[14]
BaY_4TiO_9	$Ba_{0.167}Y_{0.667}Ti_{0.167}O_{1.5}$	89	Probe Structure[14]
$Ba_2Y_2Ti_4O_{13}$	$Ba_{0.25}Y_{0.25}Ti_{0.5}O_{1.625}$	44	Probe Structure[14]
BaY_2TiO_6	$Ba_{0.25}Y_{0.5}Ti_{0.25}O_{1.5}$	69	Probe Structure[14]
**$Ba_2Y_6O_{11}$**	**$Ba_{0.25}Y_{0.75}O_{1.375}$**	54	Probe Structure
**$BaTi_3O_7$**	**$Ba_{0.25}Ti_{0.75}O_{1.75}$**	19	Probe Structure
$Ba_4Y_2Ti_6O_{19}$	$Ba_{0.333}Y_{0.167}Ti_{0.5}O_{1.583}$	76	Probe Structure[14]
$Ba_2Y_2Ti_2O_9$	$Ba_{0.333}Y_{0.333}Ti_{0.333}O_{1.5}$	70	Probe Structure[14]
$Ba_4Y_6Ti_2O_{17}$	$Ba_{0.333}Y_{0.5}Ti_{0.167}O_{1.417}$	96	Probe Structure[14]
$Ba_4Y_2Ti_2O_{11}$	$Ba_{0.5}Y_{0.25}Ti_{0.25}O_{1.375}$	62	Probe Structure[14]
$Ba_3Y_2TiO_8$	$Ba_{0.5}Y_{0.333}Ti_{0.167}O_{1.333}$	61	Probe Structure[14]
**$Ba_2Y_2O_5$**	**$Ba_{0.5}Y_{0.5}O_{1.25}$**	45	Probe Structure
**$Ba_8Y_2Ti_2O_{15}$**	**$Ba_{0.667}Y_{0.167}Ti_{0.167}O_{1.25}$**	42	Probe Structure
**Ba_3TiO_5**	**$Ba_{0.75}Ti_{0.25}O_{1.25}$**	32	Probe Structure
**$Ba_6Y_2O_9$**	**$Ba_{0.75}Y_{0.25}O_{1.125}$**	38	Probe Structure
$Ba_2Ti_9O_{20}$	$Ba_{0.182}Ti_{0.818}O_{1.818}$	4	Known[S41]
BaY_2O_4	$Ba_{0.333}Y_{0.667}O_{1.333}$	0	Known[S42]
TiO_2	TiO_2	0	Known[S43, S44]
$Ba_6Y_2Ti_4O_{17}$	$Ba_{0.5}Y_{0.167}Ti_{0.333}O_{1.417}$	2	Known[12]
Y_2O_3	$YO_{1.5}$	0	Known[S45]
$Y_2Ti_2O_7$	$Y_{0.5}Ti_{0.5}O_{1.75}$	6	Known[S46]
TiY_2O_5	$Ti_{0.333}Y_{0.667}O_{1.667}$	0	Known[S47]
Ba_2TiO_4	$Ba_{0.667}Ti_{0.333}O_{1.333}$	0	Known[S48]
$Ba_4Ti_{13}O_{30}$	$Ba_{0.235}Ti_{0.765}O_{1.765}$	0	Known[S49]
$Ba_6Ti_{17}O_{40}$	$Ba_{0.261}Ti_{0.739}O_{1.739}$	7	Known[S50]
BaO	BaO	0	Known[S51]
$BaTi_2O_5$	$Ba_{0.333}Ti_{0.667}O_{1.667}$	13	Known[S52]
$BaTi_4O_9$	$Ba_{0.2}Ti_{0.8}O_{1.8}$	8	Known[S50]
$BaTi_5O_{11}$	$Ba_{0.167}Ti_{0.833}O_{1.833}$	4	Known[S53]
$BaTiO_3$	$Ba_{0.5}Ti_{0.5}O_{1.5}$	0	Known[S54]

Compositions highlighted in bold indicate new compositions in this work. Type (final column) indicates whether the composition is from probe structures or a previously reported phase. The energy from the convex hull is computed with DFT in this work.

Table S2 Figures of merit for different machine learning regression models[S55] predicting the thermal conductivity, sorted by the coefficient of determination R^2. MSE denotes the mean squared error. Random forest and neural networks perform similarly, and outperform other models by a considerable margin.

Model	R^2	MSE
Random forest	0.66	18.3
Multilayer perceptron 64x2	0.65	18.9
k-nearest neighbours	0.55	24.4
Ridge	0.55	24.3
AdaBoost	0.40	32.4
Support vector machine	0.39	32.9
Elastic net	0.31	37.0
Lasso	0.26	39.9
Linear	0.11	47.6

Table S3 Calculated values of lattice thermal conductivity for some known materials in the Y^{3+} − Ba^{2+} − Ti^{4+} − O^{2-} phase field, taken from the TEDesignLab dataset featured in the work of Gorai *et al.*[17] See Figure S1 for a visual comparison. Quaternary materials in the face and ternaries along the edges of the phase field were not included in the training data, but were included in the testing set to help assess performance of the final random forest and neural network models, which have a root mean squared error (RMSE) of 4.3 W m^{-1} K^{-1} across the entire dataset. The RMSE across the data subset below is 5.8 W m^{-1} K^{-1} for the final random forest models and 5.1 W m^{-1} K^{-1} for the final neural network models. When compared to the performance on the entire dataset, the similar RMSE in this data subset suggests the model is appropriate to use in this phase field, and the lower RMSE for the neural network models suggests they are more appropriate than the random forest models in this case and are thus used in this work to justify experimental prioritization (Figure 1).

Composition	$\kappa_{lattice}$ (W m^{-1} K^{-1}) [calculated]	κ (W m^{-1} K^{-1}) [predicted] random forest	κ (W m^{-1} K^{-1}) [predicted] neural network
TiO_2	7.6	24.4	26.2
Ti_2O_3	36.9	23.1	29.0
$Y_2Ti_2O_7$	26.2	17.3	17.2
Y_2O_3	15.9	18.5	16.7
BaO	3.0	5.7	6.1
BaO_2	0.9	7.5	5.7
$Ba_3Y_4O_9$	12.4	12.5	7.3
BaY_2O_4	11.8	14.4	11.6
Ba_2TiO_4	5.7	11.0	9.7
$BaTi_2O_5$	13.0	16.9	12.0
$BaTi_4O_9$	16.3	17.3	17.5

Table S4 Parameters from fitting the variation in unit cell volume as a function of temperature.

	V_0	V_1	V_2	V_3	V_4	R^2
$Ba_{10}Y_6Ti_4O_{27}$	391.096(14)	–3.2(7) × 10^{-3}	8.1(9) × 10^{-5}	–2.3(4) × 10^{-7}	2.8(7) × 10^{-10}	0.9998
$Ba_6Y_2Ti_4O_{17}$	1791.81(8)	1.8(8) × 10^{-3}	1.4(2) × 10^{-4}	–1.7(4) × 10^{-7}	-	0.9994

Polynomial terms used to describe the temperature dependence of unit cell volumes of $Ba_{10}Y_6Ti_4O_{27}$ (Figure 5d) and $Ba_6Y_2Ti_4O_{17}$ (Figure S20) through $V_0 + V_1T + V_2T^2 + V_3T^3 + V_4T^4$, obtained from non-linear fitting.

Table S5 Parameters for the phenomenological modelling of κ_{latt} for $Ba_{10}Y_6Ti_4O_{27}$.

Parameter	Value	Origin
θ_{D1} (K)	290(2)	Obtained from fitting heat capacity (Figure S7, Table S9)
θ_{D2} (K)	800(10)	Obtained from fitting heat capacity (Figure S7, Table S9)
θ_E (K)	95.5(5)	Obtained from fitting heat capacity (Figure S7, Table S9)
v_s (m s^{-1})	2204(45)	Obtained from experimental measurement of Grüneisen parameter
A (s^4 m^{-1})	1.42 × 10^{-46}	Point-defect scattering calculated via Eq. S10
B (s^2 m^{-1})	1.2(1) × 10^{-16}	Umklapp scattering contribution extracted via modelling $\kappa(T)$
C (s^2 m^{-1} K^{-1})	8.1(2) × 10^{-15}	Extended defect contribution extracted via modelling $\kappa(T)$
D (m^{-1})	9.9(1) × 10^{10}	Resonant scattering contribution extracted via modelling $\kappa(T)$
Γ (Dimensionless)	0.3(2)	Extracted via modelling $\kappa(T)$
l_{min1} (Å)	6.0(2)	Extracted via modelling $\kappa(T)$
l_{min2} (Å)	2.5(2)	Extracted via modelling $\kappa(T)$

Parameters used in modelling the lattice thermal conductivity of $Ba_{10}Y_6Ti_4O_{27}$ and their origin. Parameters A, B, C, D and l_{mini} are described in Eq. S10-13.

Table S6 Compositions used in the Y^{3+} – Sr^{2+} – Ti^{4+} – O^{2-} phase field stability calculations.

Composition	Normalized Composition	Energy from convex hull (meV atom^{-1}) from DFT- this work	Source of structural model
$Ti_6Y_2O_{15}$	$Ti_{0.75}Y_{0.25}O_{1.875}$	58	Probe Structure[13]
$Ti_2Y_6O_{13}$	$Ti_{0.25}Y_{0.75}O_{1.625}$	58	Probe Structure[13]
$Sr_2Ti_8Y_2O_{21}$	$Sr_{0.167}Ti_{0.667}Y_{0.167}O_{1.75}$	58	Probe Structure[13]
$SrTi_3Y_2O_{10}$	$Sr_{0.167}Ti_{0.5}Y_{0.333}O_{1.667}$	37	Probe Structure[13]
$Sr_2Ti_4Y_6O_{19}$	$Sr_{0.167}Ti_{0.333}Y_{0.5}O_{1.583}$	80	Probe Structure[13]
$SrTiY_4O_9$	$Sr_{0.167}Ti_{0.167}Y_{0.667}O_{1.5}$	103	Probe Structure[13]
$Sr_2Ti_4Y_2O_{13}$	$Sr_{0.25}Ti_{0.5}Y_{0.25}O_{1.625}$	125	Probe Structure[13]
$SrTiY_2O_6$	$Sr_{0.25}Ti_{0.25}Y_{0.5}O_{1.5}$	79	Probe Structure[13]
$Sr_2Y_6O_{11}$	$Sr_{0.25}Y_{0.75}O_{1.375}$	34	Probe Structure[13]
$SrTi_3O_7$	$Sr_{0.25}Ti_{0.75}O_{1.75}$	43	Probe Structure[13]
$Sr_4Ti_6Y_2O_{19}$	$Sr_{0.333}Ti_{0.5}Y_{0.167}O_{1.583}$	76	Probe Structure[13]
$Sr_2Ti_2Y_2O_9$	$Sr_{0.333}Ti_{0.333}Y_{0.333}O_{1.5}$	119	Probe Structure[13]
$Sr_4Ti_2Y_6O_{17}$	$Sr_{0.333}Ti_{0.167}Y_{0.5}O_{1.417}$	85	Probe Structure[13]
$Sr_4Ti_2Y_2O_{11}$	$Sr_{0.5}Ti_{0.25}Y_{0.25}O_{1.375}$	129	Probe Structure[13]
$Sr_3TiY_2O_8$	$Sr_{0.5}Ti_{0.167}Y_{0.333}O_{1.333}$	59	Probe Structure[13]
$Sr_2Y_2O_5$	$Sr_{0.5}Y_{0.5}O_{1.25}$	40	Probe Structure[13]
$Sr_8Ti_2Y_2O_{15}$	$Sr_{0.667}Ti_{0.167}Y_{0.167}O_{1.25}$	93	Probe Structure[13]
Sr_3TiO_5	$Sr_{0.75}Ti_{0.25}O_{1.25}$	13	Probe Structure[13]
$Sr_6Y_2O_9$	$Sr_{0.75}Y_{0.25}O_{1.125}$	57	Probe Structure[13]
$Sr_6Ti_4Y_2O_{17}$	$Sr_{0.5}Ti_{0.333}Y_{0.167}O_{1.417}$	121	Probe Structure[13]
SrY_2O_4	$Sr_{0.333}Y_{0.667}O_{1.333}$	4	Known[S56]
Y_2O_3	$YO_{1.5}$	0	Known[S45]
$Y_2Ti_2O_7$	$Y_{0.5}Ti_{0.5}O_{1.75}$	0	Known[S46]
TiY_2O_5	$Ti_{0.333}Y_{0.667}O_{1.667}$	0	Known[S47]
Sr_2TiO_4	$Sr_{0.667}Ti_{0.333}O_{1.333}$	0	Known[S57]
$Sr_3Ti_2O_7$	$Sr_{0.6}Ti_{0.4}O_{1.4}$	0	Known[S58]
$Sr_4Ti_3O_{10}$	$Sr_{0.57}Ti_{0.43}O_{1.43}$	0	Known[S58]
SrO	SrO	0	Known[S59]
$SrTiO_3$	$Sr_{0.5}Ti_{0.5}O_{1.5}$	0	Known[S60]
TiO_2	TiO_2	0	Known[S43, S44]

Type (final column) indicates whether the composition is from probe structures or a previously reported phase. The energy from the convex hull is computed with DFT in this work.

Table S7 Summary of physical properties for new $Ba_{10}Y_6Ti_4O_{27}$.

**$Ba_{10}Y_6Ti_4O_{27}$**				
**α_V (K^{-1})**	$-8.2(17) \times 10^{-6} + 4.1(4) \times 10^{-7}\,T - 1.8(3) \times 10^{-9}\,T^2 + 2.8(7) \times 10^{-12}\,T^3$			
**$V_{300\ K}$ (Å^3)**	393.330(11)			
**Z**	2			
**m_r (g mol^{-1})**	2530.174			
**B_T (GPa)**	103.5			
**N (m^{-3})**	5.974×10^{28}			
**v_s (m s^{-1}) at 300 K**	2204(45)			
**T (K)**	**C_P (J mol^{-1} K^{-1})**	**C_V (J mol^{-1} K^{-1})**	**α_V (10^{-6} K^{-1})**	**γ**
12	12.60	12.60	-3.55	-3.43
20	45.67	45.67	-0.69	-0.18
40	172.9	172.97	5.56	0.39
60	318.83	318.75	10.66	0.41
80	449.26	449.04	14.73	0.40
100	556.92	556.53	17.92	0.39
120	640.65	640.04	20.35	0.39
140	707.18	706.34	22.17	0.38
160	764.04	762.96	23.50	0.38
180	815.60	814.28	24.48	0.37
200	864.64	863.09	25.24	0.36
220	907.98	906.17	25.93	0.35
240	941.53	939.44	26.66	0.35
260	963.03	960.60	27.59	0.35
280	978.47	975.62	28.84	0.36
300	1000.08	996.65	30.54	0.38

Temperature dependence of heat capacity (C_P), volumetric thermal expansion coefficient (α_V), and Grüneisen parameter (γ) for $Ba_{10}Y_6Ti_4O_{27}$, along with parameters used in extraction of speed of sound (v_s). B_T is the bulk modulus which is estimated from the elastic constants using DFT, N is the atomic number density, and m_r is the molar mass.

Table S8 Summary of physical properties for known $Ba_6Y_2Ti_4O_{17}$.

$Ba_6Y_2Ti_4O_{17}$				
α_V (K^{-1})	$1.0(19) \times 10^{-6} + 1.64(19) \times 10^{-7}\ T - 2.8(6) \times 10^{-10}\ T^2$			
$V_{300\ K}$ (Å^3)	1800.88(10)			
Z	4			
m_r (g mol^{-1})	1465.4			
B_T (GPa)	106.7			
N (m^{-3})	6.441×10^{28}			
v_s (m s^{-1}) at 300 K	4443(21)			
**T (K)**	**C_P (J mol^{-1} K^{-1})**	**C_V (J mol^{-1} K^{-1})**	**α_V (10^{-6} K^{-1})**	**γ**
12	3.40	3.40	2.99	25.31
20	23.62	23.61	4.30	5.25
40	105.62	105.55	7.58	2.07
60	192.35	192.15	10.87	1.63
80	267.79	267.33	14.15	1.52
100	329.59	328.72	17.43	1.53
120	381.35	379.87	20.71	1.57
140	428.04	425.71	23.99	1.62
160	469.62	466.18	27.27	1.69
180	505.21	500.36	30.56	1.76
200	533.85	527.24	33.84	1.85
220	557.78	549.03	37.12	1.95
240	580.18	568.86	40.40	2.05
260	604.42	590.08	43.68	2.14
280	630.00	612.14	46.96	2.22
300	641.56	619.65	50.25	2.35

Temperature dependence of heat capacity (C_P), volumetric thermal expansion coefficient (α_V), and Grüneisen parameter (γ) for $Ba_6Y_2Ti_4O_{17}$, along with parameters used in extraction of speed of sound (v_s). B_T is the bulk modulus which is estimated from the elastic constants using DFT, N is the atomic number density, and m_r is the molar mass.

Table S9 Parameters from modelling the heat capacity of $Ba_{10}Y_6Ti_4O_{27}$.

θ_{D1} (K)	θ_{D2} (K)	N_{D1}	N_{D2}	θ_{E1} (K)	θ_{E2} (K)	N_{E1}	N_{E2}	Linear term (J mol^{-1} K^{-2})
290(2)	800(10)	21.62(8)	20.92(8)	95.5(5)	54.3(4)	4.47(9)	0.235(5)	0.025(1)

Parameters obtained from fitting experimental heat capacity data of $Ba_{10}Y_6Ti_4O_{27}$. θ_{Di} and θ_{Ei} correspond to Debye and Einstein temperatures, respectively. N_{Di} and N_{Ei} are the number of atoms per formula unit with Debye temperature θ_{Di} and Einstein temperature θ_{Ei}, respectively.

References

[S1] G. Kresse, J. Furthmuller, *Phys. Rev. B* **1996**, *54*, 11169-11186.
[S2] J. P. Perdew, K. Burke, M. Ernzerhof, *Phys. Rev. Lett.* **1996**, *77*, 3865-3868.
[S3] G. Kresse, D. Joubert, *Phys. Rev. B*, **1999**, *59*, 1758-1775.
[S4] S. P. Ong, W. D. Richards, A. Jain, G. Hautier, M. Kocher, S. Cholia, D. Gunter, V. L. Chevrier, K. A. Persson, G. Ceder, *Comput. Mater. Sci.* **2013**, *68*, 314-319.
[S5] H. Peng, Z.-H. Yang, J. P. Perdew, *J. Sun, Phys. Rev. X*, **2016**, *6*, 041005.
[S6] R. Hill, *Proc. Phys. Soc., London, Sect. A*, **1952**, *65*, 349-355.
[S7] L. Ward, A. Dunn, A. Faghaninia, N. E. R. Zimmermann, S. Bajaj, Q. Wang, J. Montoya, J. Chen, K. Bystrom, M. Dylla, K. Chard, M. Asta, K. A. Persson, G. J. Snyder, I. Foster, A. Jain, *Comput. Mater. Sci.* **2018**, *152*, 60-69.
[S8] L. Ward, A. Agrawal, A. Choudhary, C. Wolverton, *npj Comput. Mater.* **2016**, *2*, 16028
[S9] F. Pedregosa, G. Varoquaux, A. Gramfort, V. Michel, B. Thirion, O. Grisel, M. Blondel, P. Prettenhofer, R. Weiss, V. Dubourg, *J. Mach. Learn. Res.* **2011**, *12*, 2825-2830.
[S10] M. Abadi, P. Barham, J. Chen, Z. Chen, A. Davis, J. Dean, M. Devin, S. Ghemawat, G. Irving, M. Isard, *in 12th {USENIX} Symposium on Operating Systems Design and Implementation ({OSDI} 16)*, **2016**, pp. 265-283.
[S11] D. P. Kingma, J. Ba, *Preprint at https://arxiv.org/abs/1412.6980*, **2014**.
[S12] B. Meredig, E. Antono, C. Church, M. Hutchinson, J. Ling, S. Paradiso, B. Blaiszik, I. Foster, B. Gibbons, J. Hattrick-Simpers, A. Mehta, L. Ward, *Mol. Syst. Des. Eng.* **2018**, *3*, 819-825
[S13] L. Palatinus, C. A. Correa, G. Steciuk, D. Jacob, P. Roussel, P. Boullay, M. Klementova, M. Gemmi, J. Kopecek, M. C. Domeneghetti, F. Camara, V. Petricek, *Acta Cryst. B*, **2015**, *71*, 740-751.
[S14] V. Petříček, V. Eigner, M. Dušek, A. Čejchan, *Z. Kristallogr. - Cryst. Mater.* **2016**, *231*, 301-312.
[S15] V. Petříček, M. Dušek, L. Palatinus, *Z. Kristallogr. - Cryst. Mater.* **2014**, *229*, 345-352
[S16] J. Potter, J. E. Parker, A. R. Lennie, S. P. Thompson, C. C. Tang, J. Appl. Crystallogr. **2013**, *46*, 826-828.
[S17] L. Palatinus, G. Chapuis, *J. Appl. Crystallogr.* **2007**, *40*, 786-790.
[S18] C. Baerlocher, L. B. McCusker, L. Palatinus, *Z. Kristallogr.* **2007**, *222*, 47-53.
[S19] M. Evain, V. Petříček, *Ferroelectrics* **2004**, *305*, 43-48.
[S20] V. Pecharsky, P. Zavalij, *Fundamentals of powder diffraction and structural characterization of materials*, Springer, **2008**.
[S21] E. S. Toberer, L. L. Baranowski, C. Dames, *Annu. Rev. Mater. Res.* **2012**, *42*, 179-209.
[S22] T. H. K. Barron, *Ann. Phys.* **1957**, *1*, 77-90.
[S23] C. Borgnakke, R. E. Sonntag, *Fundamentals of Thermodynamics*, Wiley, **2013**, pp. 557-608.
[S24] C. Kittel, *Introduction to solid state physics*, Wiley, **2005**.
[S25] M. Tachibana, E. Takayama-Muromachi, *Phys. Rev. B*, **2009**, *79*, 100104.
[S26] W. A. Phillips, A. Anderson, *Amorphous solids: low-temperature properties*, Springer, **1981**.

[S27] L. M. Daniels, S. N. Savvin, M. J. Pitcher, M. S. Dyer, J. B. Claridge, S. Ling, B. Slater, F. Corà, J. Alaria, M. J. Rosseinsky, *Energy Environ. Sci.* **2017**, *10*, 1917-1922.
[S28] B. Sattibabu, A. K. Bhatnagar, S. S. Samatham, D. Singh, S. Rayaprol, D. Das, V. Siruguri, V. Ganesan, *J. Alloys Compd.* **2015**, *644*, 830-835.
[S29] G. A. Slack, S. Galginaitis, *Phys. Rev.* **1964**, *133*, A253-A268.
[S30] R. O. Pohl, *Phys. Rev. Lett.* **1962**, *8*, 481-483.
[S31] Y. P. Joshi, Phys. *Status Solidi B*, **1979**, *95*, 317-324.
[S32] R. A. Richardson, S. D. Peacor, C. Uher, F. Nori, *J. Appl. Phys.* **1992**, *72*, 4788-4791.
[S33] P. G. Klemens, *Proc. Phys. Soc., London, Sect. A,* **1955**, *68*, 1113-1128.
[S34] S. Mukhopadhyay, D. S. Parker, B. C. Sales, A. A. Puretzky, M. A. McGuire, L. Lindsay, *Science*, **2018**, *360*, 1455-1458.
[S35] M. T. Agne, R. Hanus, G. J. Snyder, *Energy Environ. Sci.* **2018**, *11*, 609-616.
[S36] X. Kuang, J. B. Claridge, T. Price, D. M. Iddles, M. J. Rosseinsky, *Inorg. Chem.* **2008**, *47*, 8444-8450.
[S37] L. Miranda, K. Boulahya, D. C. Sinclair, M. Hernando, A. Varela, J. M. González-Calbet, M. Parras, *J. Mater. Chem.* **2012**, *22*, 15092-15103.
[S38] Y. Bando, Y. Sekikawa, H. Yamamura, Y. Matsui, *Acta Cryst. A*, **1981**, *37*, 723-728.
[S39] K. Luo, M. A. Hayward, *Inorg. Chem.* **2012**, *51*, 12281-12287.
[S40] A. Colville, *Acta Cryst. B*, **1970**, *26*, 1469-1473.
[S41] G. D. Fallon, B. M. Gatehouse, *J. Solid State Chem.* **1983**, *49*, 59-64
[S42] G. A. Costa, M. Ferretti, M. L. Fornasini, E. A. Franceschi, G. L. Olcese, *Powder Diffr.* **1989**, *4*, 24-25.
[S43] M. Horn, C. F. Schwerdtfeger, E. P. Meagher, *Z. Kristallogr. - Cryst. Mater.* **1972**, *136*, 273-281.
[S44] W. H. Baur, A. A. Khan, *Acta Cryst. B*, **1971**, *27*, 2133-2139.
[S45] M. G. Paton, E. *N. Maslen, Acta Cryst.* **1965**, *19*, 307-310.
[S46] B. V. Kumar, R. Velchuri, V. R. Devi, G. Prasad, B. Sreedhar, C. Bansal, M. Vithal, *J. Appl. Phys.* **2010**, *108*, 044906.
[S47] W. G. Mumme, A. D. Wadsley, *Acta Cryst. B*, **1968**, *24*, 1327-1333.
[S48] K. K. Wu, I. D. Brown, *Acta Cryst. B*, **1973**, *29*, 2009-2012
[S49] E. Tillmanns, *Inorg. Nucl. Chem. Lett.* **1971**, *7*, 1169-1171.
[S50] W. Hofmeister, E. Tillmanns, W. H. Baur, *Acta Crystallogr. Sect. C-Cryst. Struct. Commun.* **1984**, *40*, 1510-1512.
[S51] L. G. Liu, *J. Appl. Phys.* **1971**, *42*, 3702-3704.
[S52] E. Tillmanns, *Acta Cryst. B*, **1974**, *30*, 2894-2896.
[S53] E. Tillmanns, *Acta Cryst. B*, **1969**, *25*, 1444-1452.
[S54] R. H. Buttner, E. N. Maslen, *Acta Cryst. B*, **1992**, *48*, 764-769
[S55] S. Russell, and P. Norvig, *Artificial Intelligence: A Modern Approach 4th edn*, Prentice Hall, **2020**.
[S56] H. Müller-Buschbaum, Z. Anorg. *Allg. Chem.* **1968**, *358*, 138-146.
[S57] K. Kawamura, M. Yashima, K. Fujii, K. Omoto, K. Hibino, S. Yamada, J. R. Hester, M. Avdeev, P. Miao, S. Torii, T. Kamiyama, *Inorg. Chem.* **2015**, *54*, 3896-3904.
[S58] S. N. Ruddlesden, P. Popper, *Acta Cryst.* **1958**, *11*, 54-55.
[S59] J. Bashir, R. T. A. Khan, N. M. Butt, G. Heger, *Powder Diffr.* **2002**, *17*, 222-224.
[S60] R. J. Nelmes, G. M. Meyer, J. Hutton, *Ferroelectrics* **1978**, *21*, 461-462.

Author Contributions

The research project was conceived by C.C., M.J.P., M.S.D., G.R.D., J.B.C. and M.J.R. C.C. carried out probe structure calculations. M.W.G. and M.M. developed machine learning models for property prediction. C.C. performed the synthetic work with assistance from R.F. C.C. solved the crystal structure with supervision from J.B.C. and D.P. C.D. and M.Z. performed electron microscopy. O.P and D.P. carried out precession electron diffraction tomography. C.A.M. assisted with measurement of low-temperature synchrotron X-ray diffraction data. M.W.G. and L.M.D. processed the materials to density for property measurement with assistance from G.G. L.M.D., Q.G. and J.A. performed all property measurements and modelled physical property data. M.J.R. directed the research with assistance from M.J.P., L.M.D. and T.D.M. M.J.R. wrote the first draft. All authors contributed to the development of the manuscript and discussion throughout the project.
